# Characterization of a humanized mouse model of Duchenne muscular dystrophy to support the development of genetic medicines

**DOI:** 10.1242/dmm.052182

**Published:** 2025-10-17

**Authors:** Kara Braunreiter, Amber Kempton, Maria Katherine Mejia-Guerra, Andrew Murray, Stephen Baine, Kaitlin Adegboye, Alex Haile, Suruchi Jai Kumar Ahuja, Alessandra Fedoce, Chang Liu, Peter Burch, Ami Meda Kabadi

**Affiliations:** Sarepta Therapeutics Inc., Cambridge, MA 02142, USA

**Keywords:** Duchenne muscular dystrophy, Dystrophin, Gene editing, Mouse model, Preclinical

## Abstract

Duchenne muscular dystrophy (DMD) is a rare, progressive neuromuscular disease resulting from *DMD* variants, leading to loss of functional dystrophin. To evaluate human-targeted genetic medicines for functional dystrophin restoration, humanized genetic models containing the full human locus are required. This study characterized the hDMDΔ52/*mdx* mouse model previously reported by Pickar-Oliver and colleagues. Genomic characterization confirmed complete *DMD* duplication with identical exon 52 deletion junctions on both copies. Histological analysis showed increased diaphragm fibrosis and skeletal muscle central nuclei in hDMDΔ52/*mdx* mice versus hDMD/*mdx* controls. hDMDΔ52/*mdx* mice demonstrated reduced tibialis anterior specific force, decreased skeletal muscle fiber diameter, decreased resistance to eccentric contraction-induced damage and cardiac defects. Multiple serum biomarkers of disease were identified. Using a CRISPR/Cas9 gene-editing strategy to restore human functional dystrophin protein expression, detectable dystrophin expression in the heart and skeletal muscle and increased resistance to injury in the tibialis anterior muscle were observed. In summary, hDMDΔ52/*mdx* mice display multiple physiological and functional deficits associated with DMD pathology, which can be restored by human-targeted therapy, confirming the suitability of this model for developing human-targeted genetic medicines.

## Teaser

**Summary:** The humanized genetic model of Duchenne muscular dystrophy (DMD), the hDMDΔ52/*mdx* mouse, displays molecular and functional deficits of DMD, confirming its suitability for developing human genetic medicines for treatment of DMD.

## INTRODUCTION

Duchenne muscular dystrophy (DMD) is a rare, progressive and debilitating neuromuscular disease resulting from pathogenic variants in the *DMD* gene that lead to reduced levels of functional dystrophin (Aartsma-Rus et al., 2006). Because dystrophin protects against contraction-induced injury in muscle (Blake et al., 2002; Zhao et al., 2016), reduced levels of dystrophin protein in DMD result in progressive and irreversible functional loss of skeletal and cardiac muscle, culminating in loss of ambulation by the early teens and death before 30 years of age (Asher et al., 2020; Birnkrant et al., 2018; Broomfield et al., 2021; Nascimento Osorio et al., 2019).

Although a large number of preclinical models are available for DMD, spanning non-mammalian and mammalian systems, mouse and dog models are the most widely employed owing to their ease of use and ability to recapitulate human disease phenotypes, respectively (van Putten et al., 2020; Wasala et al., 2020). These models have not only been used to identify molecular mechanisms underpinning key aspects of DMD pathology, they have also furthered advances in therapeutic development. The *mdx* mouse model contains a nonsense mutation in exon 23 of the mouse *Dmd* gene and is the most widely used preclinical DMD model (Bulfield et al., 1984; Willmann et al., 2009), providing a good balance of throughput and recapitulation of measurable phenotypic deficits seen in humans (e.g. central myonuclei, fibrosis and cardiomyopathy) (van Putten et al., 2020). However, murine phenotypes are generally less severe than those observed in large-animal models and humans (Engelbeen et al., 2020; Signorelli et al., 2023; van Putten et al., 2019).

Despite species differences in disease severity, preclinical mouse models have been critical for analyzing various dystrophin restoration strategies for DMD, including exon skipping (Dunckley et al., 1998; Gan et al., 2022; Wilton et al., 1999), gene therapy (Phelps et al., 1995; Potter et al., 2021) and gene editing (Long et al., 2016; Nelson et al., 2019). Although murine and human dystrophin are 91% homologous at the protein level, they only share 5% homology at the genomic sequence level. Emerging therapeutic modalities, such as gene editing and antisense oligonucleotides (ASOs), are designed to target specific gene sequences, thereby necessitating the use of mouse models harboring humanized *DMD* gene sequences to test human drug candidates. Towards this end, multiple humanized genetic models of DMD have been developed, the first of which was hDMD mice containing a 2.3-megabase insertion of the human dystrophin locus into chromosome 5 (‘t Hoen et al., 2008). hDMD/*mdx* mice were created by backcrossing hDMD mice to mice deficient in mouse dystrophin (*mdx*). Although this model allowed for the assessment of target engagement by human-directed therapeutics, hDMD/*mdx* mice contain the wild-type human *DMD* gene, which precludes its use in evaluating functional deficits associated with DMD. Building off the work of ‘t Hoen et al. (2008), three groups independently developed different dystrophic humanized models from hDMD/*mdx* mice by creating single exon deletions in the human copy of the *DMD* gene, thereby producing frameshifts and downstream nonsense mutations to create human dystrophin-deficient mouse models (Pickar-Oliver et al., 2021; Veltrop et al., 2018; Young et al., 2017). These groups focused on creating mutations that can be corrected via exon skipping strategies that address large patient populations based on the known mutation spectrum of patients with DMD (Leckie et al., 2024). Veltrop et al. (2018) used transcription activator-like effector nucleases (TALENs) in conjunction with homology-directed repair to create an exon 52 deletion (del52hDMD/*mdx* mice). Young et al. (2017) and Pickar-Oliver et al. (2021) used CRISPR/Cas9-based strategies to delete exon 45 (hDMDdel45/*mdx* mice) or exon 52 (hDMDΔ52/*mdx* mice), respectively. A partially humanized mouse model (ΔD50;h51KI model) was also developed by replacing the mouse exon 51 with human exon 51 and deleting mouse exon 50, rendering this chimeric mouse/human dystrophin out of frame and thereby dystrophin null (Zhang et al., 2022). Restoration of dystrophin expression through human targeted therapeutic intervention has been demonstrated in all of these models (Pickar-Oliver et al., 2021; Veltrop et al., 2018; Young et al., 2017; Zhang et al., 2022).

Although the pathology of the *mdx* mouse has been thoroughly characterized in the field (Coulton et al., 1988; Pastoret and Sebille, 1995; Potter et al., 2021; Sharp et al., 2011; Turk et al., 2005), it is important to phenotypically characterize these new humanized mouse derivatives to confirm their dystrophic presentation. Therefore, this study aimed to characterize the previously unreported skeletal and cardiac deficits and serum biomarkers in the hDMDΔ52/*mdx* mouse model reported by Pickar-Oliver et al. (2021). To date, this model has primarily been used to demonstrate dystrophin protein restoration following genetic intervention (Pickar-Oliver et al., 2021). Building on the prior work that described moderate respiratory pathology in this model (Roger et al., 2024), this study evaluated the performance of male hDMDΔ52/*mdx* mice in various physiological assessments [tibialis anterior (TA) physiology and histology, electrocardiogram (ECG)] and functional assessments (grip strength and rotarod), as well as serum biomarkers, compared with those in age-matched hDMD/*mdx* controls. The impact of functional dystrophin restoration by gene editing with CRISPR/Cas9 was also examined to further support the use of this model for clinical development of genetic medicines for DMD.

## RESULTS

### Genomic characterization of hDMDΔ52/mdx mouse

**Fig. 1.**
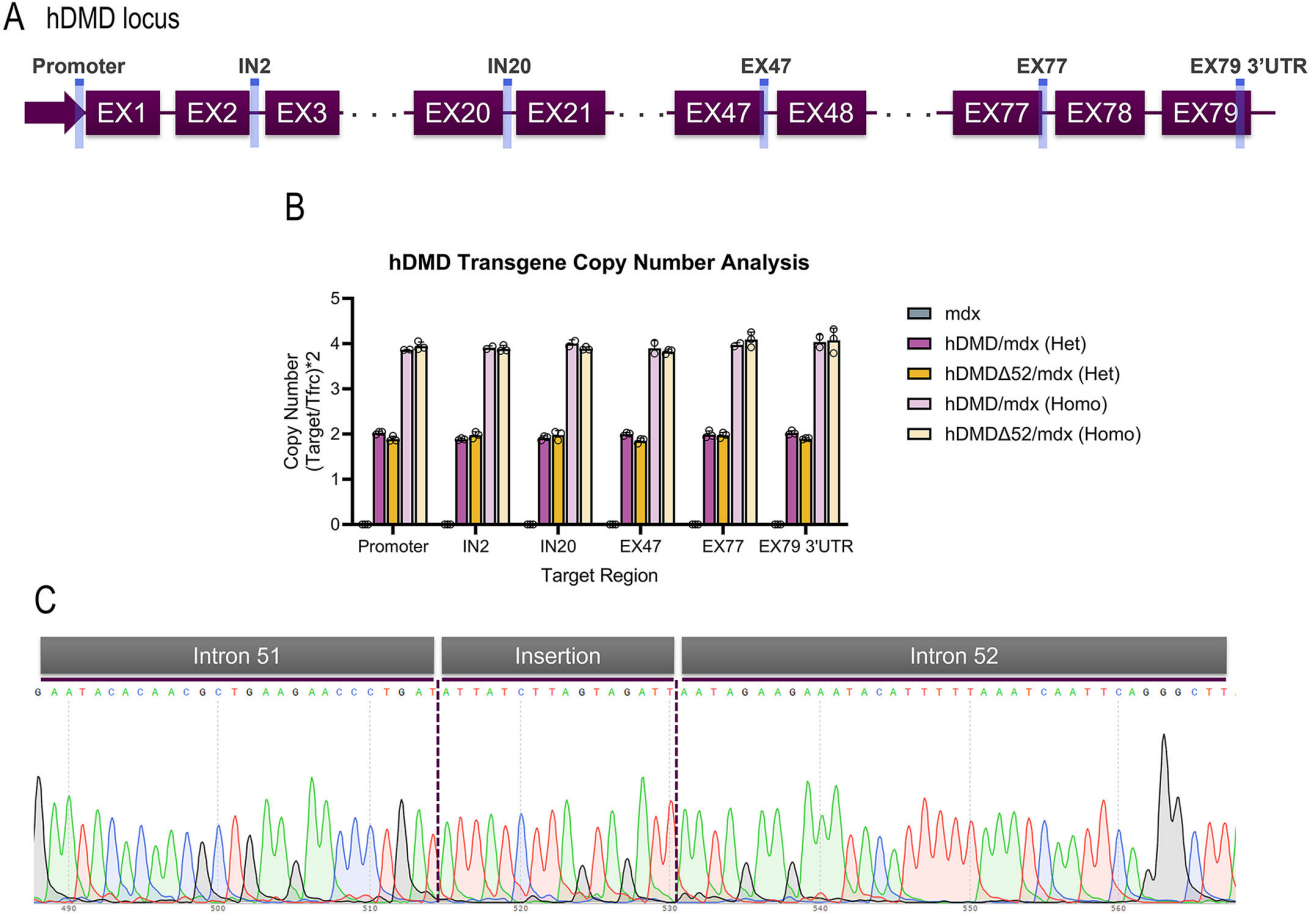
**Genomic characterization of hDMD/*mdx* and hDMDΔ52/*mdx* mice.** (A,B) Digital droplet PCR assays were designed targeting various regions of the *DMD* transgene, highlighted in blue (A), and were used to assess the *DMD* copy number at each location (B). Each dot represents the copy number results for an individual animal in the corresponding region assessed. EX, exon; Het, heterozygote; Homo, homozygote; IN, intron; UTR, untranslated region. (C) The predicted intron 51-intron 52 junction was PCR amplified from hDMDΔ52/*mdx* genomic DNA. Sanger sequencing identified a 16-bp insertion at the deletion site.

Although it was previously reported that the parental hDMD (hDMD/*mdx*) mouse generated by ‘t Hoen and collaborators (‘t Hoen et al., 2008; Yavas et al., 2020) contains a tail-to-tail duplication event (Yavas et al., 2020), this duplication event has not yet been evaluated in the hDMDΔ52/*mdx* model developed by Pickar-Oliver et al. (2021). Towards this end, we designed digital droplet PCR (ddPCR) probes spanning from the entire *DMD* locus, including 2 kb of sequence upstream and downstream of the annotated *DMD* gene (Fig. 1A). ddPCR definitively detected two copies in heterozygous hDMD/*mdx* and hDMDΔ52/*mdx* mice, and four copies in homozygous hDMD/*mdx* and hDMDΔ52/*mdx* mice, suggesting a complete duplication (Fig. 1B).

To further characterize the genomic composition of the hDMD/*mdx* control line (‘t Hoen et al., 2008) and the hDMDΔ52/*mdx* disease line (Pickar-Oliver et al., 2021), we performed long-read whole-genome sequencing (WGS) (Table S1). A copy number variation analysis confirmed that there is complete duplication of the entire *DMD* locus, including 2 kb of sequence upstream and downstream of the annotated *DMD* sequence in the hDMD/*mdx* parental line and that the duplication is maintained in the daughter hDMDΔ52/*mdx* line generated by Pickar-Oliver et al. (2021) (Figs S1 and S2).

Insertion of the *DMD* transgene was originally reported to mouse chromosome 5 (‘t Hoen et al., 2008). To similarly determine the *DMD* insertion location, we aligned the WGS data to the Hygromycin cassette sequence and yeast genome (as a proxy for the YAC sequence used to generate the hDMD transgenic mouse) and further used those reads to map against the mouse genome. This analysis pointed to regions with mapped reads common to the transgenic lines but absent in the control *mdx* line, with the most likely position for the insertion overlapping with the 5qG2 band towards the end of chromosome 5. We pinpointed the insertion around coordinates chr5:133,745,208-133,829,563, which closely agrees with the recent work of Chey et el. (2024) (Table S2).

Next, the Δ52 deletion was characterized using structural variation analysis. A unique 309 bp deletion event was detected spanning human chrX:31327616-31327924, which overlaps exon 52, as annotated for the main isoform expressed in muscle (NCBI transcript ID, NM_004006.3; ENSMBL transcript ID, ENST00000357033.9). The observation of a unique deletion event suggests that the identical edit was made on both copies during the creation of this mouse (Fig. S3). To further understand the region in which the deletion occurred, we generated a *de novo* assembly from the WGS data. We recovered one single contig containing the full *DMD* region, including at least 2 kb upstream and downstream of the genic region, from each of the hDMD/*mdx* and hDMDΔ52/*mdx* lines and none from the control *mdx* line (Fig. S4). A search of the sequences corresponding to the 79 annotated exons for the main isoform expressed in muscle (i.e. Dp427m) revealed that exon 52 and flanking regions in the adjacent introns are present in the assembled loci for the parent hDMD/*mdx* line (Fig. S4A) and absent from the hDMDΔ52/*mdx* line, as expected (Fig. S4B,C). Follow-up Sanger sequencing revealed a 16-bp insertion in the intronic region between the guide RNA (gRNA) cut sites (Fig. 1C), consistent with a previous report (Robinson-Hamm, 2018). The same insertion was confirmed in the *de novo* assembled *DMD* loci for each of the hDMDΔ52/*mdx* samples (Fig. S3B,C). An additional search of the 16-bp insertion sequence directly in the reads that map to the *DMD* allele showed similar counts to the neighbor exons in the hDMDΔ52/*mdx* mice and zero count in the hDMD/*mdx* mice, suggesting that this same 16-bp insertion can be found on both copies of the hDMDΔ52 allele (Table S3).

### Histological analysis of hDMDΔ52/*mdx* tissues

**Fig. 2.**
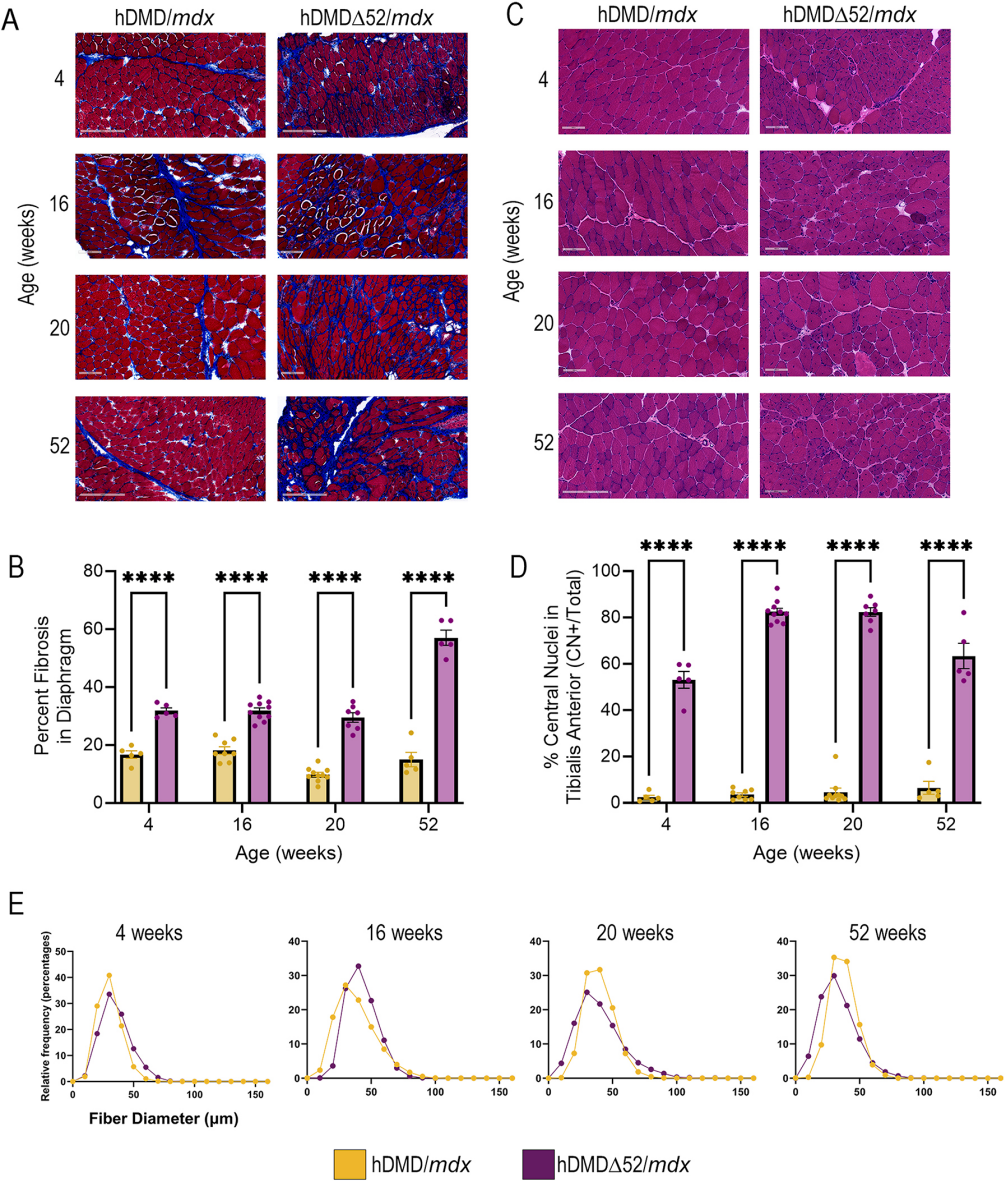
**Histological analysis of hDMDΔ52/*mdx* tissues reveals cellular defects.** (A) Representative images of Masson's trichrome staining of the diaphragm at 4, 16, 20 and 52 weeks of age. Scale bars: 200 μm at 4 and 52 weeks; 100 μm at 16 and 24 weeks. (B) Quantification of the percentage of fibrosis in the diaphragm of hDMD/*mdx* and hDMDΔ52/*mdx* mice. ****P<0.0001, two-way ANOVA with Šídák's multiple comparisons test. (C) Representative images of Hematoxylin and Eosin (H&E) staining in the tibialis anterior (TA) muscle at 4, 16, 20 and 52 weeks of age. Scale bars: 200 μm for hDMD/*mdx* at 52 weeks; 100 μm for other images. (D) Quantification of central nuclei at the indicated ages. ****P<0.0001, two-way ANOVA with Šídák's multiple comparisons test. (E) TA muscle fiber diameter at the indicated ages. Data represent *n*=5-10 individual animals per group. Šídák's multiple comparisons test.

Next, the hDMDΔ52/*mdx* mice were assessed for histological outcomes across skeletal muscles and heart, which are tissues known to have clinical manifestations in patients with DMD (Deconinck and Dan, 2007). Because DMD primarily affects young boys, all animals assessed were male to eliminate any impact of sex on the outcomes. At all ages assessed, Masson's trichrome staining showed increased fibrosis in the diaphragm of hDMDΔ52/*mdx* mice compared with that in hDMD/*mdx* mice (Fig. 2A,B). However, minimal fibrosis was apparent in the heart at later timepoints (Fig. S5). Fiber diameter in the TA muscle of hDMDΔ52/*mdx* mice varied considerably, with hDMDΔ52/*mdx* mice showing a broader distribution of fiber diameters and reduced mean fiber diameter at older ages compared to hDMD/*mdx* controls at similar ages (16 weeks, 39.8 vs 42.7 µm; 20 weeks, 41.2 vs 41.6 µm; and 52 weeks, 34.1 vs 37.4 µm, respectively) (Fig. 2C,E). The same trends were observed in the gastrocnemius (GAS) and triceps brachii (TRI) muscles (Fig. S6A-C). Furthermore, indicative of the high turnover rate of dystrophic muscle fibers, hDMDΔ52/*mdx* mice had increased central nuclei at all ages in several muscles, including the TA (4 weeks, 53.1% vs 2.4%; 16 weeks, 82.5% vs 3.6%; 20 weeks, 82.4% vs 4.7%; 52 weeks, 63.4% vs 6.5% at 52 weeks) (Fig. 2C,D), GAS (4 weeks, 39.8% vs 1.3%; 16 weeks, 74.1% vs 2.2%; 20 weeks, 70.6% vs 6.6%; 52 weeks, 57.6% vs 12.9%) and TRI (4 weeks, 56.4% vs 3.3%; 16 weeks, 76.6% vs 3.3%; 20 weeks, 69.7% vs 6.8%; 52 weeks, 70.0% vs 14.1%) (Fig. S6A,D,E), compared to those in the hDMD/*mdx* control strain. This finding is consistent with numerous other mouse DMD models (Yucel et al., 2018), including reports of 50% central nuclei in other humanized mouse models of DMD (Veltrop et al., 2018) and up to 80% central nuclei in *mdx* mice (Coulton et al., 1988). The hDMDΔ52/*mdx* strain described here shows a range of central nuclei from 39.8% to 82.5%, depending on the age and tissue examined.

### hDMDΔ52/*mdx* mice display functional deficits characteristic of DMD

**Fig. 3.**
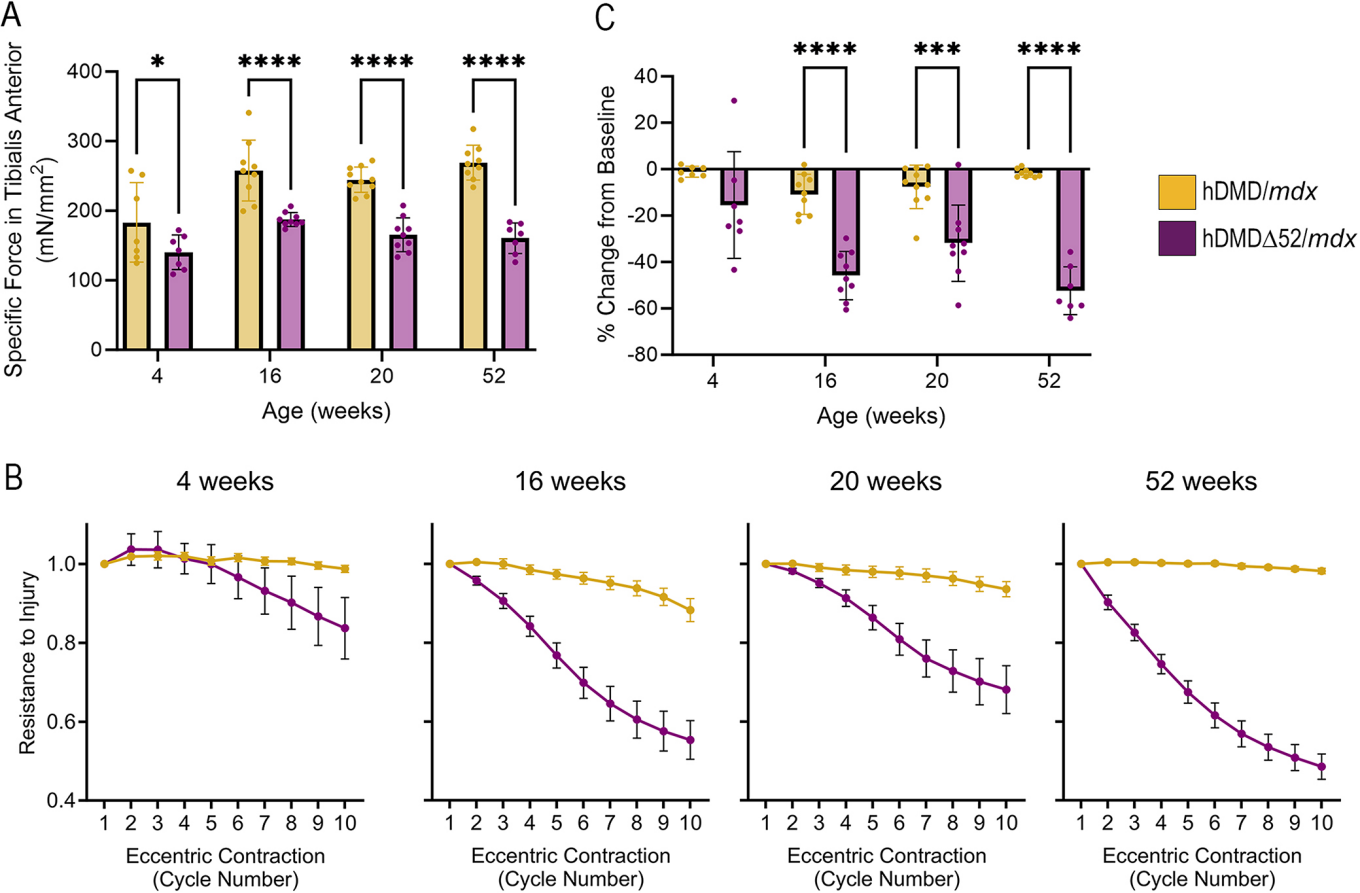
**TA dysfunction in hDMDΔ52/*mdx* mice.** (A) TA specific force at the indicated timepoints. (B) TA resistance to injury by eccentric contraction. (C) Percentage drop after ten eccentric contractions in the TA. Data represent *n*=8-10 individual animals per group. *P<0.05, ***P<0.001, ****P<0.0001, two-way ANOVA.

**Fig. 4.**
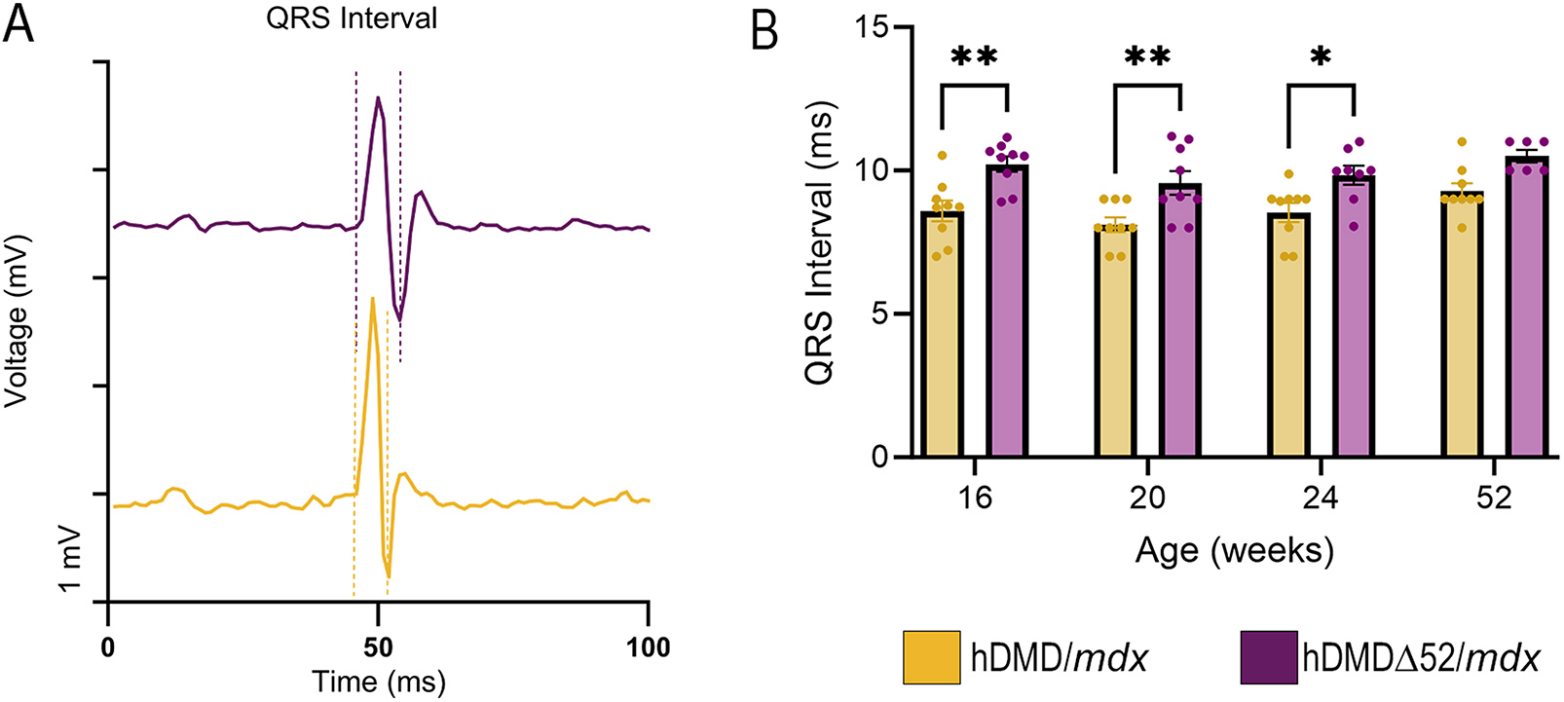
**Physiological deficits in heart rhythm in hDMDΔ52/*mdx* mice.** (A) Representative electrocardiogram analyses at 20 weeks. (B) Mean QRS interval length is elongated in hDMDΔ52/*mdx* mice compared to that in hDMD/*mdx* mice. Data represent *n*=6-10 individual animals per group. *P<0.05, **P<0.01, two-way ANOVA.

The phenotypic hallmark of DMD is muscle damage and weakness due to contraction (Deconinck and Dan, 2007). As shown in Fig. 3A, the specific force (normalized to cross-sectional area of the tissue) of the TA was reduced in hDMDΔ52/*mdx* mice compared to that in age-matched hDMD/*mdx* control animals at 4 weeks (22% reduction), 16 weeks (27% reduction), 20 weeks (33% reduction) and 52 weeks (41% reduction) of age. This is consistent with the reported reduction of 29% in TA specific force in *mdx* mice compared to that in wild-type controls (Sharp et al., 2011). Resistance to damage by eccentric contractions was also reduced in the TA of hDMDΔ52/*mdx* mice starting at 16 weeks of age (Fig. 3B,C), consistent with only an average 25% maintenance of maximal force observed in *mdx* mice (Sharp et al., 2011). hDMDΔ52/*mdx* mice also display cardiac rhythm defects as detected by ECG under anesthesia (Fig. 4). Many DMD mouse models have not recapitulated cardiac defects that are associated with human disease, whereas hDMDΔ52/*mdx* mice displayed an increased QRS interval (Fig. 4), which does mimic human disease (van Putten et al., 2020). Other aspects of cardiac rhythm were unaffected (Fig. S7). Mild deficits in grip strength and rotarod were observed at select timepoints (Fig. S8).

### Serum biomarkers

**Fig. 5.**
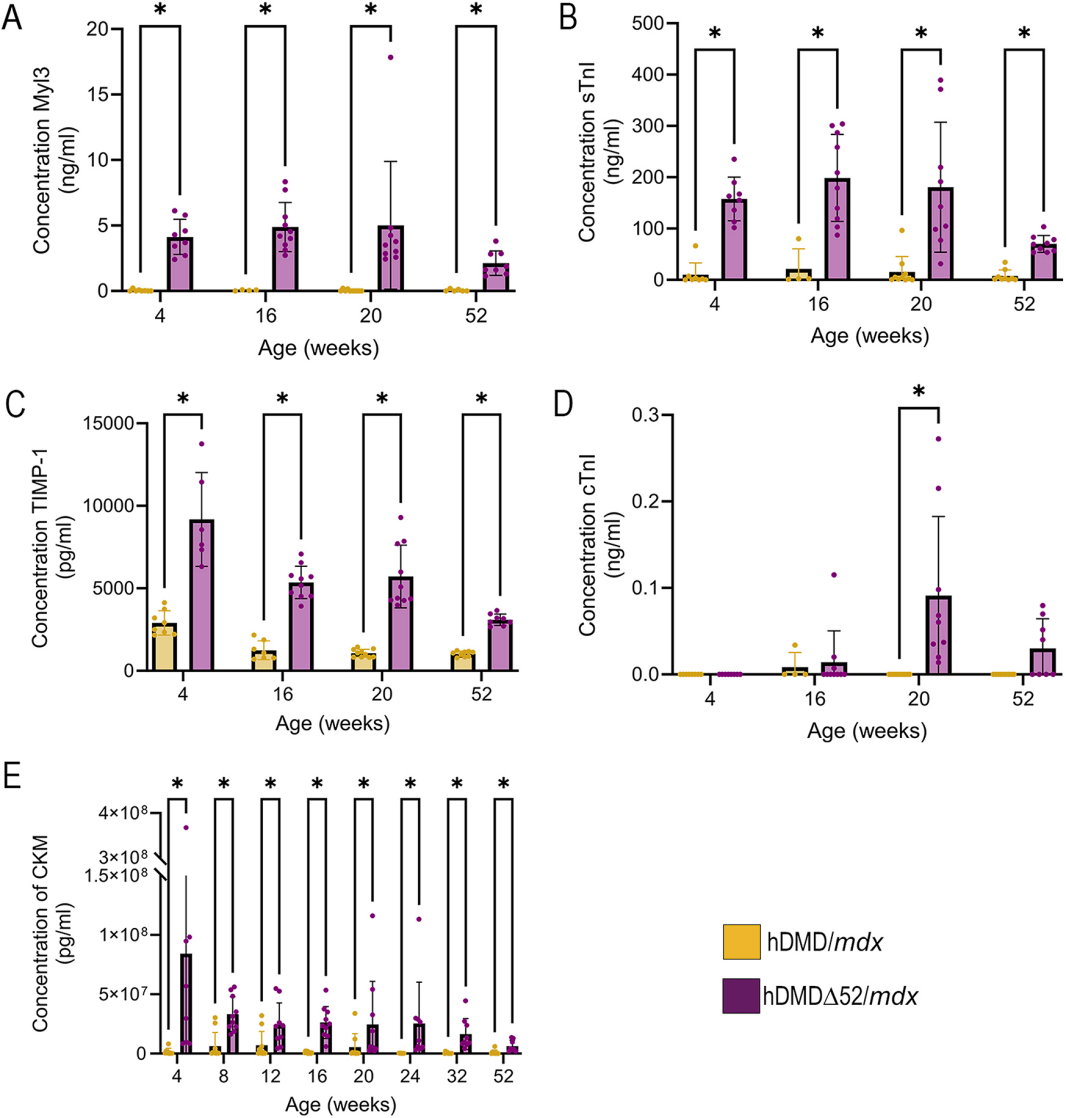
**Elevated serum biomarkers of muscle and cardiac injury in hDMDΔ52/*mdx* mice.** (A-E) Quantification of Myl3 (A), sTnI (B), TIMP-1 (C), cTnI (D) and CKM (E). Data represent *n*=8-10 individual animals per group. *P<0.05, Mann–Whitney test for nonparametric data.

Serum biomarkers for skeletal muscle and cardiac injury were assessed at various ages in both hDMD/*mdx* and hDMDΔ52/*mdx* animals through in-life serum collections prior to assessment of functional or physiological outcomes. Markers of skeletal muscle injury include skeletal troponin I (sTnI; also known as TNNI1/2), myosin light chain 3 (Myl3), fatty acid binding protein 3 (FABP3), muscle-type creatine kinase (CKM) and matrix metalloproteinase 9 (MMP-9). sTnI and Myl3 are myofibrillar proteins involved in the contraction of muscles (Schiaffino and Reggiani, 1996). The release of myofibrillar proteins into the circulation increases in response to muscle damage; thus, these biomarkers are expected to increase in response to muscle damage in models of muscular dystrophy (Burch et al., 2015; Pavis et al., 2021). As expected, serum levels of Myl3 and sTnI were elevated in hDMDΔ52/*mdx* animals at most ages (Fig. 5A,B). FABP3 and CKM are secreted by muscle fibers into the blood upon muscle damage (Burch et al., 2015), particularly in cases of muscular dystrophy. Levels of FABP3 were elevated in some hDMDΔ52/*mdx* mice at 4 and 16 weeks of age (Fig. S9A). Levels of CKM were significantly elevated in hDMDΔ52/*mdx* mice, compared to those in hDMD*/mdx* mice, at all ages (Fig. 5E), consistent with reports of elevated serum FABP3 and CKM levels in *mdx* mice (Glesby et al., 1988).

Cardiac biomarkers included cardiac troponin I (cTnI; also known as TNNI3), tissue inhibitor of metalloproteinase 1 (TIMP-1), FABP3 and MMP-9 (Niebroj-Dobosz et al., 2015). Serum levels of TIMP-1 were elevated in hDMDΔ52/*mdx* mice compared to those in hDMD/*mdx* controls at all ages (Fig. 5C). cTnI is a biomarker of coronary artery disease. The levels of cTnI were below fit-curve range in the hDMD/*mdx* animals but were elevated in numerous hDMDΔ52/*mdx* animals at later time points, as shown in Fig. 5D, indicating a notable difference between the two strains of mice. MMP-9 is secreted from both skeletal muscle and cardiac muscle into the extracellular matrix, where it exacerbates skeletal muscle and cardiac muscle phenotypes in DMD by contributing to increased inflammation and muscle damage and reduced skeletal and cardiac muscle function (Kherif et al., 1999; Li et al., 2009; Ogura et al., 2014). No differences in MMP-9 serum levels were detected between the two strains (Fig. S9B). However, it is possible that our hDMD/*mdx* mice retain higher levels of MMP-9 than wild-type animals owing to the *mdx* background of the strain; thus, differences between hDMDΔ52/*mdx* and hDMD/*mdx* mice could not be observed (Dahiya et al., 2011a,b; Kherif et al., 1999; Li et al., 2009).

### Functional restoration of dystrophin expression in hDMDΔ52/*mdx* mice through therapeutic intervention

**Fig. 6.**
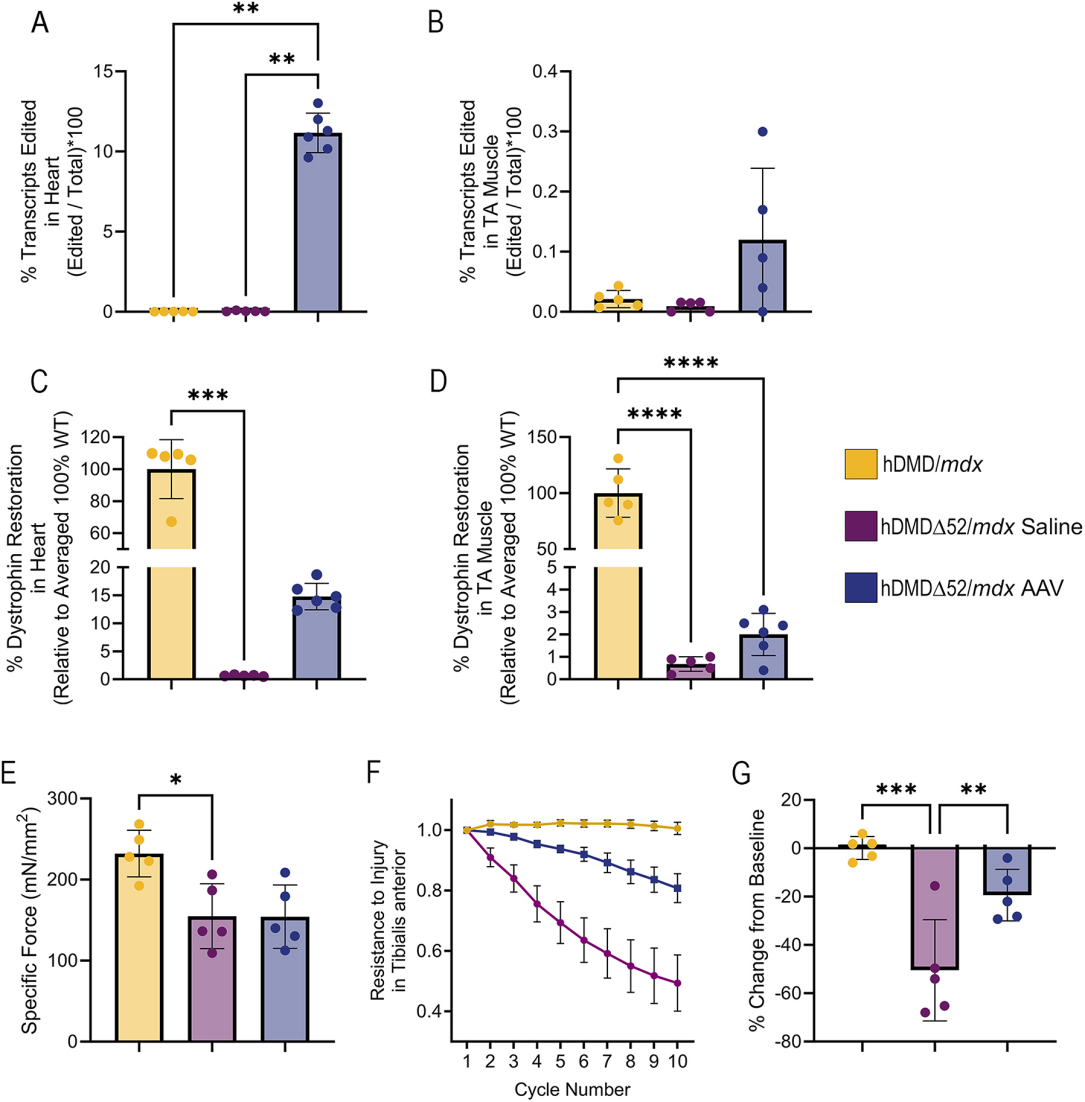
**Functional restoration of the hDMDΔ52/*mdx* model following therapeutic intervention with gene editing at 12 weeks post-treatment.** (A,B) Percentage edited transcripts in the heart (A) and TA (B). **P<0.01, Kruskal–Wallis test with Dunn's post-hoc analysis for non-normal data. (C,D) Dystrophin protein restoration in the heart (C) and TA skeletal muscle (D). ***P<0.001, ****P<0.0001, Kruskal–Wallis Test with Dunn's post-hoc analysis for non-normal data. (E) Specific force of the TA muscle following gene editing. *P<0.05, one-way ANOVA with Dunnett's multiple comparisons to the hDMDΔ52/*mdx* saline group. (F) Resistance to TA muscle injury following gene editing. (G) Percentage decrease in force following ten eccentric contractions in TA muscle. **P<0.01, ***P<0.001, one-way ANOVA. Data represent *n*=5-6 individual animals per group.

To further support the use of this model for the development of human-targeting therapies, we next conducted a proof-of-concept study to assess whether hDMDΔ52/*mdx* deficits could be rescued through therapeutic intervention using a human-targeting CRISPR/Cas9-based gene-editing strategy to delete exons 45-55 and reframe the *DMD* gene. This is a different therapeutic strategy from that previously reported by Pickar-Oliver et al. (2021), which used a CRISPR/Cas9 homology-independent targeted integration method to integrate an exon 52 copy into the hDMDΔ52/*mdx* mouse. Here, we utilized a CRISPR/Cas9-based gene-editing strategy with two gRNAs targeting introns 44 and 55 to delete exons 45-55 and thereby produce an internally shortened, functional dystrophin protein (Gersbach et al., 2022). These two guides were packaged into AAVrh74 with a *Staphylococcus aureus* Cas9 coding sequence. Systemic administration resulted in appreciable levels of edited *DMD* transcripts in the heart and TA as measured by ddPCR (Fig. 6A,B). These edited transcripts led to 14.8% dystrophin protein restoration in the heart (Fig. 6C; Fig. S10B) and 2% dystrophin restoration in the TA (Fig. 6D; Fig. S10D) as quantified by Jess Automated Western Blot. Mice treated with gene-editing therapeutics also showed functional restoration of resistance to injury in the TA muscle at 12 weeks post-treatment, as shown by significant improvement in the retention of muscle force eccentric muscle contractions (Fig. 6E-G).

## DISCUSSION

To further the development of human genetic therapeutic approaches for DMD, mouse models containing the human target sequence(s) are required given the lack of sequence homology at DNA and RNA target sites (Aartsma-Rus and van Putten, 2019). It is also important that these models replicate human disease phenotypes to confirm the therapeutic benefit of targeted therapies. In patients with DMD and mouse models of DMD, some muscle fibers spontaneously regain dystrophin protein expression, creating a variable number of revertant fibers. Because most humanized mouse models are created by introducing an additional copy of the human *DMD* gene into the mouse genome, this may lead to increased frequency of revertant fibers. Such variability can influence the manifestation of dystrophic phenotypes, underscoring the importance of thorough and consistent characterization of these models.

Previous work has shown that a separate but comparable hDMD/del52/*mdx* model described in Veltrop et al. (2018) contains a complete duplication of the *DMD* locus and has a dystrophic phenotype characterized by CKM elevations, muscle fibers with central nuclei and some functional impairment (Veltrop et al., 2018; Yavas et al., 2020). The present study sought to characterize the previously described hDMDΔ52/*mdx* model (Pickar-Oliver et al., 2021; Roger et al., 2024), confirming that these mice also contain a comparable gene duplication owing to the shared lineage with the hDMD mouse (‘t Hoen et al., 2008). These mice also display comparable, clinically relevant DMD phenotypes to those previously reported, as well as additional phenotypic characteristics not previously evaluated, including increased diaphragm fibrosis, reduced mean fiber diameter in the TA muscle and increased central nuclei, compared to those in hDMD/*mdx* controls. hDMDΔ52/*mdx* mice also demonstrated reduced TA specific force, reduced resistance to damage by eccentric contractions and cardiac defects. Taken together, the totality of data justifies the use of the hDMDΔ52/*mdx* mouse for the continued development of human-targeting genetic medicines.

Genomic characterization of the hDMDΔ52/*mdx* mouse demonstrates a full duplication event, including 2 kb upstream and downstream of the annotated *DMD* locus, with both copies containing an identical intron 51-intron 52 junction. This suggests that both hDMDΔ52 copies likely have the capacity to be restored through therapeutic intervention. This study did not evaluate the potential complex genomic rearrangements and their effect on the various dystrophin isoforms that may result from CRISPR/Cas9 gene editing. Therefore, the genomic architecture of this mouse should be carefully considered when using it for preclinical studies, especially dose-modeling studies. Recently, Chey et al. (2024) reported a new hDMD transgenic mouse containing a single *DMD* copy. This mouse contains the wild-type human *DMD* sequence and could be used for the future development of humanized diseased models containing a single copy, thereby simplifying preclinical human-targeting therapeutic development for DMD.

Serum biomarkers of skeletal muscle injury and/or specific DMD disease milestones – including sTnI, cTnI, Myl3, TIMP-1, FABP3 and CKM (Konagaya et al., 1987; Strandberg et al., 2020) – were greater in the hDMDΔ52/*mdx* mice than in hDMD/*mdx* controls. Although diaphragm, skeletal muscle and heart fibrosis are common clinical findings in patients with DMD, the lack of fibrosis in the heart of hDMDΔ52/*mdx* animals is consistent with disease phenotypes observed in other mouse models of DMD (Quinlan et al., 2004; Roger et al., 2024; van Putten et al., 2020). Smaller body size reduces cardiac stress, which may explain the discrepancy between the mouse models and human disease phenotypes. However, QRS prolongation was noted in hDMDΔ52/*mdx* compared with hDMD/*mdx* mice. This is consistent with human patients with DMD, who have significant cardiac electrophysiological impairment (Thrush et al., 2009). It is also consistent with other DMD mouse models, including the *mdx* model, in which a prolonged QRS interval is also observed, along with other electrophysiological abnormalities, including prolonged QT interval and increased heart rate (Bostick et al., 2009; Wasala et al., 2017, 2018). Although the hDMDΔ52/*mdx* model does not have as severe phenotypes as other DMD models, this finding is unlikely to be within the normal expected variation as cardiac manifestations are well documented in DMD models. In addition, elevations in serum biomarkers of cardiac injury were observed, including elevations in FABP3, a marker of skeletal and cardiac muscle injury (Burch et al., 2015; Pritt et al., 2008), and cTnI, a marker of cardiac injury (Hammarsten et al., 2018). This is consistent with the mild and late onset of cardiac dysfunction in the background *mdx* strain (Coley et al., 2016).

Histological analysis identified a transient increase in the myofiber diameter at 4 weeks of age, followed by a gradual decline in diameter over time in the hDMDΔ/*mdx* animals compared with that in age-matched hDMD/*mdx* animals. Variability in fiber diameter is consistent with histological phenotypes observed in patients with muscular dystrophy (Ripolone et al., 2022; Wang et al., 1999) and numerous other mouse models of DMD with an *mdx* background (Yucel et al., 2018) owing to the increasing population of smaller, regenerating fibers (Watkins and Cullen, 1982). Progressive myofiber hypertrophy was previously described in the *mdx* mouse model (Massopust et al., 2020). However, unlike in the *mdx* mouse model, myofiber hypertrophy in human disease is followed by atrophy (Kornegay et al., 2012). In this way, the hDMDΔ52/*mdx* mouse model more closely mimics human disease progression and may better model myofiber pathology of human disease.

The results of this study largely align with reported findings from other humanized mouse models. As expected, the duplication event reported for the hDMD parent model (Yavas et al., 2020) was also observed in hDMDΔ52/*mdx* mice*.* hDMDΔ52/*mdx* mice and other humanized models broadly demonstrate dystrophic morphology, including centralized nuclei (Veltrop et al., 2018; Zhang et al., 2022), mononuclear infiltration (Veltrop et al., 2018; Young et al., 2017) and increased fibrosis (Young et al., 2017). CKM is the most widely reported serum biomarker (Pickar-Oliver et al., 2021; Veltrop et al., 2018; Zhang et al., 2022), which was confirmed in the hDMDΔ52/*mdx* model along with multiple additional biomarker deficits, including sTnI, cTnI, Myl3, TIMP-1 and FABP3*.* Prior humanized models also have previously demonstrated functional deficits, such as gait (Yavas et al., 2020) and muscle strength (Veltrop et al., 2018; Zhang et al., 2022), which is in alignment with the reported deficits in specific force and resistance to injury that we observed.

The present study confirms the use of hDMDΔ52/*mdx* mice for the development of human genetic therapies as expression of the human dystrophin protein can be restored through the use of human-specific gene editing-based therapies. Animals with gene-edited dystrophin expression showed increased resistance to fatigue. This is consistent with a previous study evaluating ASO-based therapies in a similar hDMD/del52/*mdx* model, which restored dystrophin expression, improved muscle function and normalized CKM levels (van Deutekom et al., 2023). Similarly, phosphorodiamidate morpholino oligomer-based exon skipping in the hDMD/del52/*mdx* model showed dystrophin restoration and improved muscle function (Lim et al., 2022). One limitation of the present study is that we did not evaluate the potential complex genomic rearrangements that can occur following CRISPR/Cas9 gene editing across the duplicated *DMD* locus. When using the hDMDΔ52/*mdx* model to develop lead clinical candidates, researchers should carefully consider the consequence of the genetic duplication on the resulting molecular and phenotypic outcomes observed. The implications of the *DMD* genetic duplication are likely to be modality and target sequence specific.

Although humanized DMD mouse models provide a valuable tool for the development of genetic medicines for the treatment of DMD, the phenotypes observed are generally quite mild compared with those observed in humans. Furthermore, animals have a normal lifespan in comparison to humans and other large-animal models of DMD. Thus, a combination of disease models is ideal to obtain a holistic understanding of each therapeutic strategy.

## Conclusions

The humanized hDMDΔ52/*mdx* model is a well-characterized model of DMD with functional, molecular and biomarker deficits that mimic DMD disease progression in humans. Gene editing also shows therapeutic efficacy in this model, validating its use for future development of human-specific genetic therapies for DMD.

## MATERIALS AND METHODS

### hDMDΔ52/*mdx* transgenic mice

hDMDΔ52/*mdx* and hDMD/*mdx* transgenic mice were obtained from Duke University. The animals were generated as previously described (Pickar-Oliver et al., 2021). The animal studies herein were conducted with adherence to the guidelines for the care and use of laboratory animals of the National Institutes of Health (NIH). All the experiments with animals were approved by the Institutional Animal Care and Use Committee (IACUC) at Sarepta Therapeutics, Inc. hDMD/*mdx* and hDMDΔ52/*mdx* mice heterozygous for the respective human *DMD* allele were assessed at various ages for histological pathology via Masson's trichrome and Hematoxylin and Eosin (H&E) staining, cardiac and skeletal injury via serum biomarkers, as well as performance in grip strength and rotarod, *in situ* physiology of the TA, and ECG (Table S4).

### Long-read sequencing for the genomic characterization of mouse lines

WGS was performed using high-throughput long-read sequencing of the hDMDΔ52/*mdx* line. For a comprehensive characterization, we included two samples of the line of interest (i.e. one sample from the hDMDΔ52/*mdx* heterozygous and one sample from hDMDΔ52/*mdx* homozygous for the respective *DMD* allele). To perform comparisons, we included a sample of the hDMD/*mdx* parental line as heterozygous for the respective *DMD* allele, and a sample of the C57BL/10-mdx (The Jackson Laboratory strain #001801) as a representative of the background, in which all the lines are maintained (Table S1).

High-molecular mass genomic DNA was obtained using a Genomic-tip 100/G kit (Qiagen, 10243). Briefly, 80 mg of frozen brain and muscle tissue from each of the mice was processed. High-molecular mass DNA samples were submitted to Azenta Life Sciences to perform PacBio library preparation and continuous long-read sequencing. The PacBio library was prepared using a SMRTbell prep kit as per the manufacturer's protocol. SMRTbell libraries were then sequenced on the PacBio Sequel II platform with v3.0 chemistry. After quality control of PacBio raw reads [LongQC v1.2.1 (Fukasawa et al., 2020)] (Table S1), PacBio raw reads were used (1) to contrast against a reference genome that included the full genome sequence of the *Mus musculus* strain C57BL/GJ (version GRCm39) plus appended genomic sequence corresponding to a fragment of the primary *Homo sapiens* genome (version GRCh38.p14) spanning the region chrX:31117222-33341388(−), which includes the annotated *hDMD* gene ±2 kb of the flanking region (NCBI gene ID, 1756; ENSMBL gene ID, ENSG00000198947); and (2) to obtain a reference-free reconstruction of the *DMD* gene by building independent *de novo* genome assemblies from each sample using hifiasm [v0.16 (Cheng et al., 2022)] and further comparing the reconstructed sequence against the already-described *DMD* gene extracted from the human reference genome to determine which contigs contained the assembled sequence.

The full set of PacBio reads was used for the alignment against the reference genome using minimap2 with option ‘-ax map-hifi’ [v2.28 (Li, 2021)], and the resulting alignment file was used as input for calling of genomic variation. Because variants of different sizes represent different challenges, we used (1) Sniffles2 [v2.6.0 (Smolka et al., 2024)] for the characterization of medium (gain and loss of ≥50 bp) variation and (2) CNVkit [0.9.12 (Olshen et al., 2011; Talevich et al., 2016)] for the characterization of large structural (gain and loss of ≥1 kb to larger genomic regions) variation. Sniffles2 was used in the presence of the reference genome to allow for the output of deletions and run in two steps as indicated for multi-sample variant calling (i.e. including the individual variant calling and next pooling of the first pass of variants for the combined and final calling) to obtain a variant calling file. CNVkit was executed in two passes. First, we built a flat reference for the *DMD* transgene and a reference from the sample C57BL/10-mdx for the mouse genome. Second, we used the ‘-method wgs’ against the concatenate references to allow for read counts to be normalized by correcting for GC content and repetitive sequences, which resulted in an estimated depth from which log2 and copy ratios could be further calculated. The circular binary segmentation algorithm was employed to infer copy number segments. The threshold values for copy number 0 were selected as the minimum log2 from the regions in the sample used as representative of the genetic background, and the threshold value for 1 was selected as the log2 value for percentile 75 of the regions in the same sample. Values for copy gains were identical to the defaults for diploid samples as specified by CNVkit.

To determine the completeness of the *DMD* assembled loci, the exon sequences for the MANE transcript (NCBI RefSeq, NM_004006.3; ENSMBL transcript ID, ENST00000357033) were used to search the assembled contigs using Seqkit and the command ‘locate -i -m 1 -p’ [v2.3.0 (Shen et al., 2016)] to allow a maximum of one mismatch of difference between the sequences. To detect the raw PacBio reads containing the insertion generated during the generation of the disease line, we used Seqkit and the command ‘locate -i -p’ [v2.3.0 (Shen et al., 2016)] for no mismatches allowed.

File format manipulation, filtering and indexing of alignments was performed using Samtools [v1.2.0 (Danecek et al., 2021)] and Bedtools [v2.27.1 (Quinlan and Hall, 2010)]. File format manipulation of read files, such as conversion between fastq and fasta formats, was performed with Seqkit [v2.3.0 (Shen et al., 2016)]. Assembled contigs containing the *DMD* gene were visualized using SnapGene (v 6.2.1). PacBio reads alignments to the reference genome were visualized using IGV [v2.11.3 (Robinson et al., 2017)]. Copy number variation results were visualized using custom scripts in python (v3.12).

### ddPCR copy number analysis

Genomic DNA was isolated from C57BL/10SCSn-DMDmdx/J mice, as well as from heterozygous and homozygous hDMD/*mdx* and hDMDΔ52/*mdx* mice, using a NucleoMag^®^ Tissue kit (Macherey-Nagel, 744300). Copy number analysis by ddPCR was performed using the QXOne Droplet Digital PCR system (Bio-Rad). Assays targeting different regions of the *DMD* transgene were multiplexed with the Tfrc copy number assay (Thermo Fisher Scientific) using ddPCR Supermix for Probes (No dUTP) and SpeI restriction enzyme (2 U/reaction). The assay targeting exon 47 was used in singleplex reactions, with accompanying singleplex reactions for Tfrc detection on the same plate. Samples were normalized to 1 ng/µl in molecular water with 3 ng DNA analyzed per 20 µl reaction. Data were analyzed using QXOne Software, setting thresholds between positive and negative droplets. Copy numbers were determined by normalizing the DMD target assay concentration to the Tfrc concentration, with Tfrc representing two copies per genome. Samples were analyzed in duplicate. Sequences for primers and probes were as follows (forward/reverse/probe, respectively): intron 2, 5′-TTTAGCTGTGCAAATCTGGAAATA-3′/5′-TGCAGCTATAAACTCTGAGGACT-3′/56-FAM/AGGAGTAAG/ZEN/CAGCAGAAGATATGGC/3IABkFQ/; intron 20, 5′-TGTAAGACGTAGATACCCGGA-3′/5′-CCTCATGCTTACGCAAACCG-3′/56-FAM/CAATTA GCG/ZEN/CTGTGTAACTACGCCAAAT/3IABkFQ/; exon 77, 5′-AGCACAGGGTTAGAGGAGGT-3′/5′-ACTGCGTGTTGGCTTCCATA-3′/56-FAM/AAGCGAGTG/ZEN/GCCTGATCCCA/3IABkFQ/; exon 79 3′ UTR, 5′-TTGTTTTGCATCCTTTTGGCGTG-3′/5′-CAGTTCTCAAATGAGCAGTGTGT-3′/56-FAM/AAGACTTCC/ZEN/TCTACCACCACACCA/3IABkFQ/; promoter region, 5′-CCCGCCTTCTCTCTCAAGTT-3′/5′-TGTCCACTGTGCTATTCTGGTT-3′/56-FAM/CCAGCATGG/ZEN/CAAGCTCTGTGA/3IABkFQ/; and exon 47, 5′-AGCTCAAGCAGACAAATCTCCA-3′/5′-CCAAAGCAAACGGTCAGGTT-3′/5HEX/TCCCCGACC/ZEN/AATGAAGCACC/3IABkFQ/. The thermal cycling conditions followed the Bio-Rad recommended Supermix for Probes (No dUTP) cycling protocol, with an annealing temperature of 60°C.

### Sanger sequencing analysis

Genomic DNA was isolated from hDMDΔ52/*mdx* heterozygous mice using the NucleoMag^®^ Tissue kit. PCR reactions were performed using NEBNext^®^ Ultra II Q5 Master Mix (New England Biolabs) and the following primers, according to the manufacturer's specifications: forward, 5′-CTGGAGGGAGGCAAAAAGGT-3′; reverse, 5′-AAGATGGCCCAGGAAGAAGC-3′. PCR products were purified using a NucleoSpin^®^ Gel and PCR Clean-up kit (Macherey-Nagel, 740609), and samples were submitted to GENEWIZ (Azenta Life Sciences) for Sanger sequencing.

### Grip strength assessments

Grip strength assessments were captured using DFE2 grip force meters (Columbus Instruments). Grip strength assessments were performed at 4, 16, 20, 24, 32 and 52 weeks of age. At each timepoint, forelimbs were assessed for visible sores or injuries prior to testing, and only those passing visual examination were tested. To obtain a grip force (g-force), rodents were allowed to grasp the pull bar with both forepaws before being pulled away from and parallel to the pull bar. This process was repeated until five grip forces for each rodent were obtained over a maximum interval of 2 min. All five grip forces were averaged to get the forelimb grip strength, which was then normalized to body weight (g).

### Rotarod assessment

Rotarod assessments were captured using a RotaMex-5 acquisition system (Columbus Instruments). Rotarod assessments were performed at 4, 16, 20, 24, 32 and 52 weeks of age. At each timepoint, forelimbs were assessed for visible sores or injuries, and only those passing visual examination were tested. Rodents were assessed four to six times per day, with a minimum 15-min break between each assessment, for 2 consecutive days. Rodents were placed on a rod moving at 4 rpm (one per lane). Once all rodents were placed, the assessment was started, and the rod speed increased by 1 rpm every 8 s until all rodents fell or 40 rpm was reached. Latency to fall was recorded in seconds, and the three highest runs between the 2 days were averaged together to obtain the average latency to fall.

### TA *in situ* physiology

*In situ* physiology assessments were performed as terminal procedures and captured using an Aurora Scientific muscle physiology acquisition system. Rodents were anesthetized with ketamine and xylazine. Once a sufficient plane of anesthesia was obtained, the TA tendon was exposed, and 4-0 silk sutures were used to secure the tendon to the lever arm of the force transducer. The knee and foot were secured to the platform under a heat lamp. Electrodes were then inserted into the sciatic nerve. With the stimulator set to 10 mN, an instant stimulation was performed to confirm proper electrode placement. After a 5-min rest, warm up protocols were run, and optimal baseline tension was determined. Force frequencies were run, followed by a 5-min wait prior to eccentric contractions being performed. Eccentric contractions performed a 10% muscle length pull to induce injury. Data were collected from both left and right TA muscles. Muscle length and weight were collected during necropsy and used to calculate the cross-sectional area, which was used for force normalization.

### ECG assessments

Subsurface ECG evaluations were performed at 16, 20, 24 and 52 weeks of age. *In vivo* surface ECGs were captured using a PowerLab acquisition system (ADInstruments). Animals were anesthetized using 1-2% isoflurane for maintenance with oxygen (1-2 l/min). Electrodes were placed sub-dermally in the corresponding limbs parallel to the limb, running from distal to proximal. The chest echo lead was placed parallel to the sternum in the direction of head to toe for the V1 position. For the V3 position, the needle was placed medially to the left midclavicular line. The V5 was placed at the anterior axillary line, caudal to the V3. A 1-min tracing was recorded in leads I and II. A 15- to 20-s tracing was recorded in leads III, aVR, aVL, aVF, V1, V3 and V5. Lead II data were analyzed using LabChart Software (ADInstruments). QTc was calculated using the Mitchell method (Mitchell et al., 1998).

### Histopathology

At necropsy, muscles (TA, GAS, TRI, diaphragm and heart) were mounted onto wooden blocks with 7% Gum Tragacanth and fresh frozen in 2-methylbutane cooled in liquid nitrogen and stored at −80°C. Tissues were cryosectioned (10-µm sections) and stained with H&E. Centrally nucleated fibers and fiber diameters were quantified using Aperio ImageScope software (Leica Biosystems). Images were first annotated to select regions of interest and remove artifacts. The percentage of myofibers with central nuclei and fiber diameter were determined by analyzing 10-μm thick cryosections of H&E-stained TA, GAS and TRI muscles, as previously described (Rodino-Klapac et al., 2007). Analysis included four fields of 20× magnification per animal per muscle, and a minimum of 250 fibers (*n*=5-10).

### Masson's trichrome staining for fibrosis

Heart and diaphragm tissue sections (10-μm sections) were cryosectioned and allowed to air dry or were stored at −40°C until staining was performed. Frozen slides were allowed to warm to ambient temperature, then fixed in 10% neutral buffered formalin for 10 min. After incubation, slides were rinsed in deionized water up to four times, followed by fixation in 95% ethanol for 10 min. Slides were then air dried for at least 15 min and placed in Bouins fixative for ∼30 min. Slides were then washed in tap water until excess stain was no longer visible, followed by a deionized water rinse. Slides were placed in Weigert's Iron Hematoxylin Solution for 5 min. After washing in running water for 5 min, slides were rinsed in distilled water before placing in Biebrich Scarlet-Acid Fuchsin for 2 min, washed in distilled water, and incubated in phosphotungstic-phosphomolybdic acid for 15 min. Slides were then placed in Aniline Blue for 5 min, washed with distilled water, and incubated in 1% aqueous acetic acid for 5 min. Slides were quickly dehydrated in two changes of 95% ethanol, followed by three changes of 100% ethanol, then transferred to xylene (Thermo Fisher Scientific, X3P-1GAL) and cleared in three changes. Glass coverslips were mounted using Cytoseal 60 medium (Electron Microscopy Sciences).

Full slide scans were prepared using an Aperio VERSA 200 Imaging System (Leica Biosystems) and analyzed for percentage collagen quantification. Analysis was performed using Aperio ImageScope software through eSlide Manager (v12.4.3.5008; Leica Biosystems). Images were first annotated to select regions of interest and to remove artifacts, such as tissue folds, off-target saining and debris. The positivity of blue chromogen (ratio of blue pixel count to red pixel count) was reported as a percentage to denote the collagen content in the annotated areas. The following equation was used to generate collagen percentage:

% collagen=blue pixel count/(red pixel count+blue pixel count)×100,

where the blue pixel count corresponded to collagen area, and the red pixel count corresponded to non-collagen muscle area.

### In-life and terminal serum processing

In-life whole blood was collected from the facial mandibular vein under isoflurane anesthesia, with blood volume not exceeding 10% of rodent total blood volume. Terminal serum was collected via cardiac puncture following anesthetization by intraperitoneal injection with ketamine/xylazine cocktail. Blood was collected in serum separator tubes, and coagulation was allowed for at least 30 min. Samples were centrifuged at a speed of at least 1780 ***g*** for 10 min. Serum was collected, snap frozen and stored at ≤−70°C before serum biomarker quantification.

### Serum biomarker quantification

Whole blood was collected, and serum was processed at 4, 8, 12, 16, 20, 24, 32 and 52 weeks of age prior to functional outcome assessments for the quantification of CKM (MM and MB isoforms), MMP-9 and TIMP-1 serum biomarkers by enzyme-linked immunosorbent assay (ELISA). Assays for MMP-9 and TIMP-1 were performed by BioAgilytix Labs (Durham, NC, USA) using the R&D Systems Quantikine^®^ ELISA Mouse Immunoassay kit for the respective biomarker and following the manufacturer's instructions. Absorbance was read using a microplate reader set to 450 nm against 570 nm. Assays for CKM (MM and MB isoforms) were performed by BioAgilytix Labs using the MSD Muscle Injury Panel (rat) 2 kit (Meso Scale Discovery).

A Muscle Injury Panel 3 (mouse) electro chemiluminescent immunoassay (Meso Scale Discovery) was used to measure skeletal and cardiac muscle injury by quantitative analysis of endogenous cTnI, FABP3, Myl3 and sTnI serum levels at 4, 8, 12, 16, 20, 24, 32 and 52 weeks of age. A standard curve for each biomarker was prepared, and all reported values were within the detection range of the standard curve. Assay plates were read using an MSD Sector Imager 120 MM plate reader (Meso Scale Discovery), and signal and mean concentration of biomarkers were determined using the Discovery Workbench (v4.0.13). Analyses were performed using Prism Software version 8.0 for Windows (GraphPad Software).

### *In vivo* gene editing-induced exon 45-55 deletion

Five- to seven-week-old hDMD/*mdx* and hDMDΔ52/*mdx* mice were injected via the tail vein with rh74.AAV solution at 2.5×10^14^ vector genomes/kg or saline control solution. The single adeno-associated virus (AAV) encoded SaCas9, sgRNAs ATATAGTAATGAAATTATTGGCAC and CATTTGTATAGAGAGGAAATGT (Gersbach et al., 2022). Animals were euthanized at 12 weeks post-administration by intraperitoneal administration of ketamine/xylazine cocktail, and tissues were fresh frozen.

### Dystrophin protein analysis

Tissues were homogenized by mechanical disruption using a Bullet Blender Tissue Homogenizer (Next Advance) in protein lysis buffer (125 mM Tris-HCl, 4 M urea, 4% SDS) supplemented with cOmplete™, Mini Protease Inhibitor Cocktail (Sigma-Aldrich). Dystrophin protein expression was analyzed on the Jess Automated Western Blot system (Bio-Techne). Protein concentrations were quantitated using a NanoDrop OneC Spectrophotometer (Thermo Fisher Scientific). A protein concentration of 0.112 mg/ml was prepared into 1× fluorescent master mix for each sample following the manufacturer's protocol using the 66-440 kDa Separation Module. Detection of dystrophin was performed using the rabbit monoclonal antibody (Abcam, ab154168) at 1:600 dilution in milk-free antibody diluent, and detection of alpha-actinin-4 loading control was performed using mouse monoclonal antibody (Abcam, ab254074) at 1:50 dilution in milk-free antibody diluent. Secondary antibodies used were donkey anti-mouse NIR (ProteinSimple, DM-009) and goat anti-rabbit HRP (ProteinSimple, DM-001) following the manufacturer’s protocol. Samples were run on the 66-440 kDa fluorescence separation plates using the 8×25 capillary cartridges. Plate layouts included the replex module and were loaded according to the manufacturer's specifications.

To calculate the percentage dystrophin restoration, the dystrophin area under the curve (AUC) was normalized to the AUC obtained for actinin-4. The average actinin-4-normalized dystrophin value from hDMD/*mdx* mice (*N*=5) was used to determine 100% dystrophin expression. For each treated animal sample, the actinin-4-normalized edited dystrophin expression was divided by the average healthy actinin-4-normalized dystrophin expression and multiplied by 100 to generate the percentage dystrophin restored.

### *DMD* transcript analysis

RNA was isolated from tissue samples using a NucleoMag^®^ RNA Kit (Macherey-Nagel) on a KingFisher Apex instrument. cDNA was generated from 70-100 ng RNA using a SuperScript VILO cDNA Synthesis Kit (Thermo Fisher Scientific) following the manufacturer's protocol with a 2-h 42°C incubation step. Transcript editing was analyzed by ddPCR using 2× ddPCR Supermix for probes (no dUTP) (Bio-Rad) and custom PrimeTime assays to quantify edited and unedited cDNA transcripts in the sample. For Δ45-55 transcript-specific edits, the custom PrimeTime Assay was designed with a HEX-labeled probe against the Δ45-55 edited cDNA sequence (forward primer, 5′-CTGAGAATTGGGAACATGC-3′; reverse primer, 5′-CATCGGAACCTTCCAGGG; probe, ACAAATGGTATCTTAAGGACCTCCAAGGTG-3′). The final primer concentration was 0.25 μM per reaction, and the final probe concentration was 0.9 μM per reaction. The reference assay used for the Δ45-55 edit-specific master mix was TaqMan Assay Hs02562862_s1 (Thermo Fisher Scientific) targeting exon 55 of the *DMD* transcript and labeled with a VIC-MGB probe. The exon 55 assay was diluted to a 1× concentration per reaction. All samples and controls were run in duplicate on a QXOne analyzer (Bio-Rad). Automated positive thresholds were generated for all samples for analysis as possible. Manual thresholds were drawn as needed.

### Statistical analysis

Statistical analysis was performed using GraphPad Prism 10.1.2 software. Data were expressed as mean±s.d. (error bars), with individual points representing individual animals. One-way or two-way ANOVA was performed for analysis of normally distributed data (histology quantification, TA physiology, ECG, protein quantification). Kruskal–Wallis test with Dunn's multiple comparisons was performed for nonparametric data (muscle fiber diameter). Mann–Whitney test was used for nonparametric data (serum biomarker quantification). No animals assessed were excluded from analysis for any outcomes reported.

## Supplementary Material

10.1242/dmm.052182_sup1 Supplementary information
