# Supplementary material for: Characterization of a humanized mouse model of Duchenne muscular dystrophy to support the development of genetic medicines
Source: Dis Model Mech. 2025 Oct 17;18(10):dmm052182. doi: 10.1242/dmm.052182 (PMC12570149; doi:10.1242/dmm.052182)
Supplement: Supplementary information [file dmm-18-052182-s1.pdf]

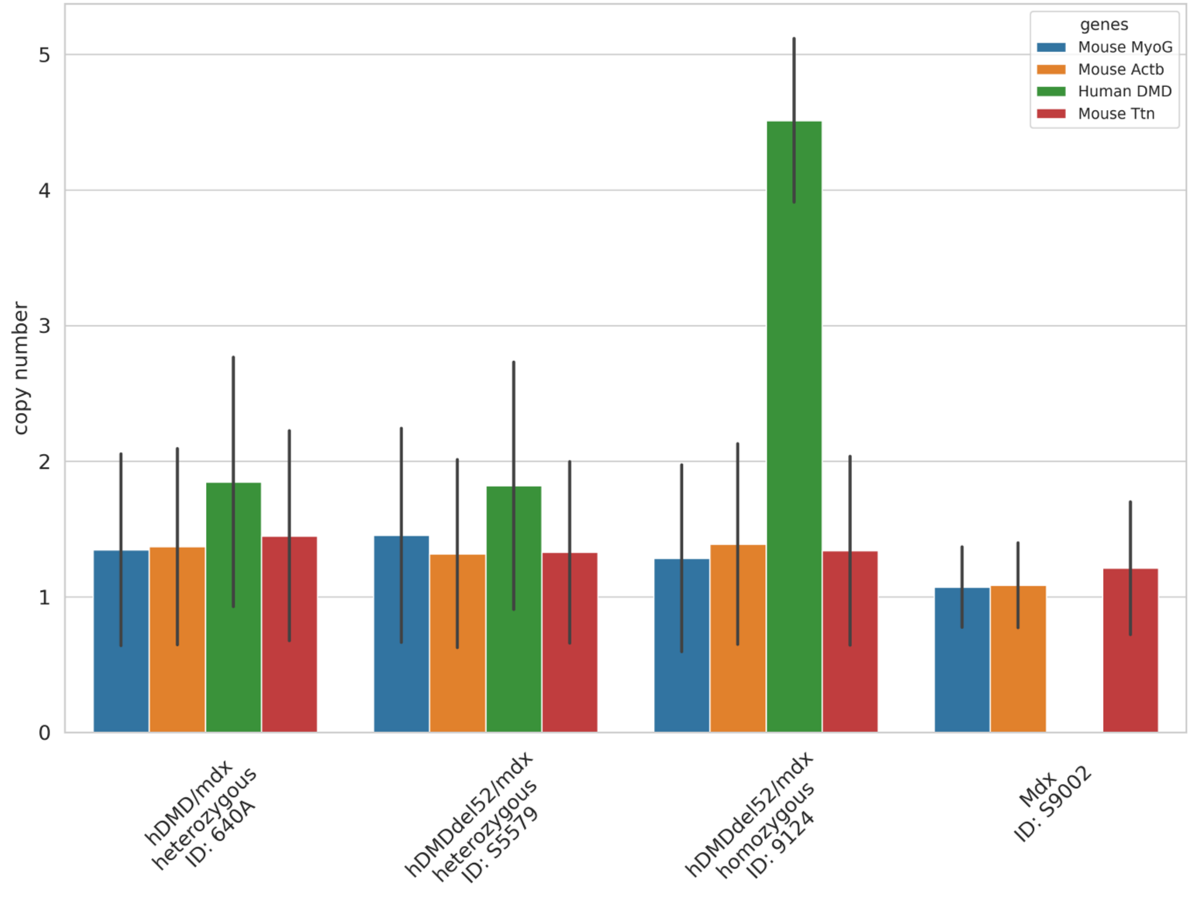

Fig. S1. Estimation of copy number variation for the *hDMD* gene and selected mouse genes. Bar plot showing the estimated copy number distribution around different genes with error bars showing the standard deviation. Analysis focused on the identification of gain/loss ≥1 kb from the WGS data. Each bar represents the integer copy number as calculated with CNVkit for a region of ~2.2 Mb centered in each of the mouse genes (*i.e.,* Actb, MyoG, Tnt) and the fully evaluated *hDMD* transgene region. As expected with a complete duplication event, most analyzed bins are around two copies in the heterozygous hDMD/*mdx* and hDMDΔ52/*mdx* samples, and around four copies in the homozygous hDMDΔ52/*mdx* sample.

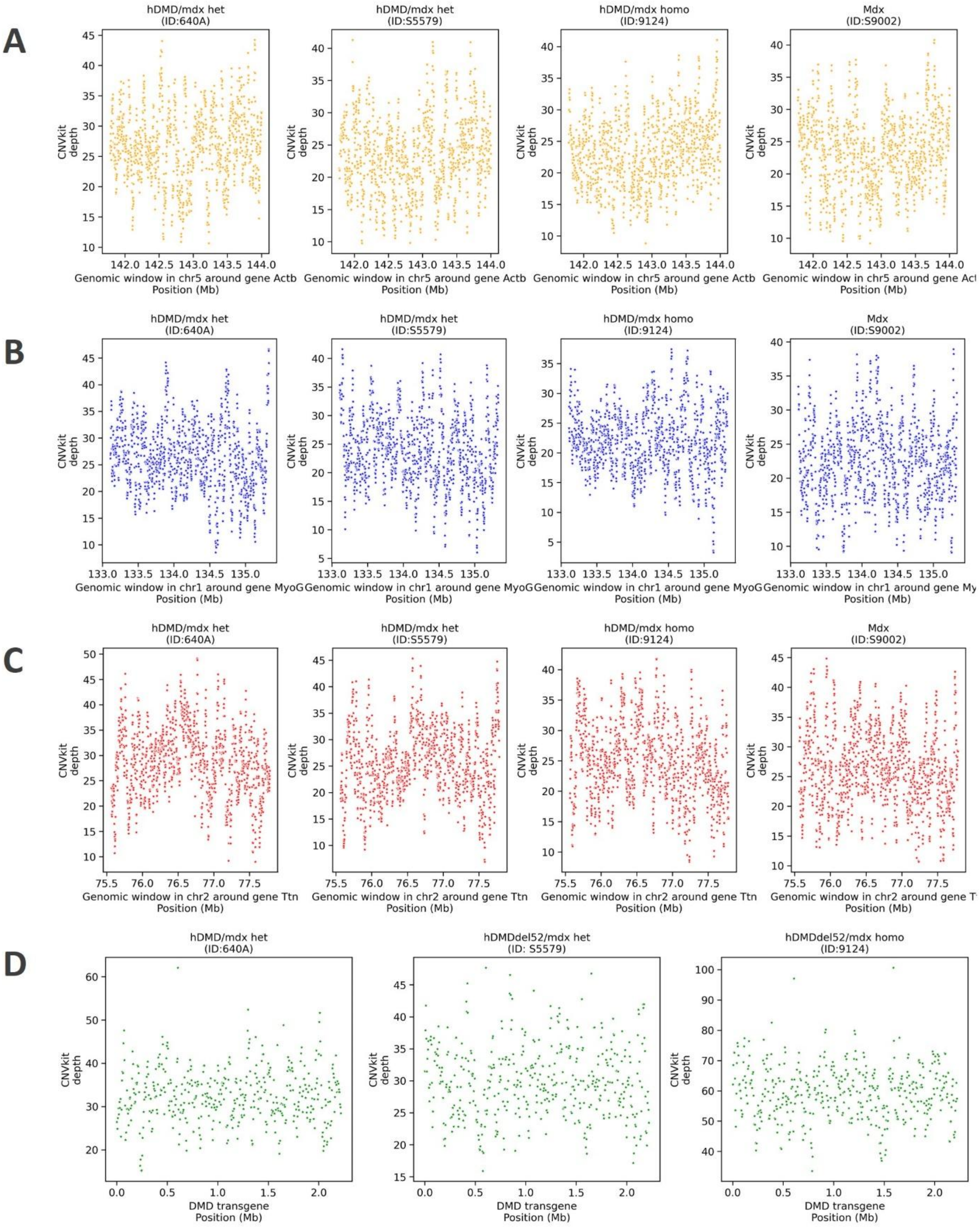
A
hDMD/mdx het
(ID:640A)
hDMD/mdx het
(ID:S5579)
hDMD/mdx homo
(ID:9124)
Mdx
(ID:S9002)
CNVkit depth
Genomic window in chr5 around gene Actb
Position (Mb)
B
hDMD/mdx het
(ID:640A)
hDMD/mdx het
(ID:S5579)
hDMD/mdx homo
(ID:9124)
Mdx
(ID:S9002)
CNVkit depth
Genomic window in chr1 around gene MyoG
Position (Mb)
C
hDMD/mdx het
(ID:640A)
hDMD/mdx het
(ID:S5579)
hDMD/mdx homo
(ID:9124)
Mdx
(ID:S9002)
CNVkit depth
Genomic window in chr2 around gene Ttn
Position (Mb)
D
hDMD/mdx het
(ID:640A)
hDMDdel52/mdx het
(ID: S5579)
hDMDdel52/mdx homo
(ID:9124)
CNVkit depth
DMD transgene
Position (Mb)

Fig. S2. Estimated read depth for human and mouse *DMD* genes. Corrected coverage by GC and repetitive regions as determined with CNVkit (v 0.9.12) for ~2 Mb around the following genes for the four evaluated mouse lines: (A) mouse *Actb* (chr5:141,778,606-144,002,773), (B) mouse *MyoG* (chr1:133,106,931-135,331,097), (C) mouse *Tnt* (chr2: 75,561,529-77,785,696), and (D) *hDMD* transgene region (for the three humanized mouse lines; the coverage for the control *mdx* mouse line is zero for the human transgene).

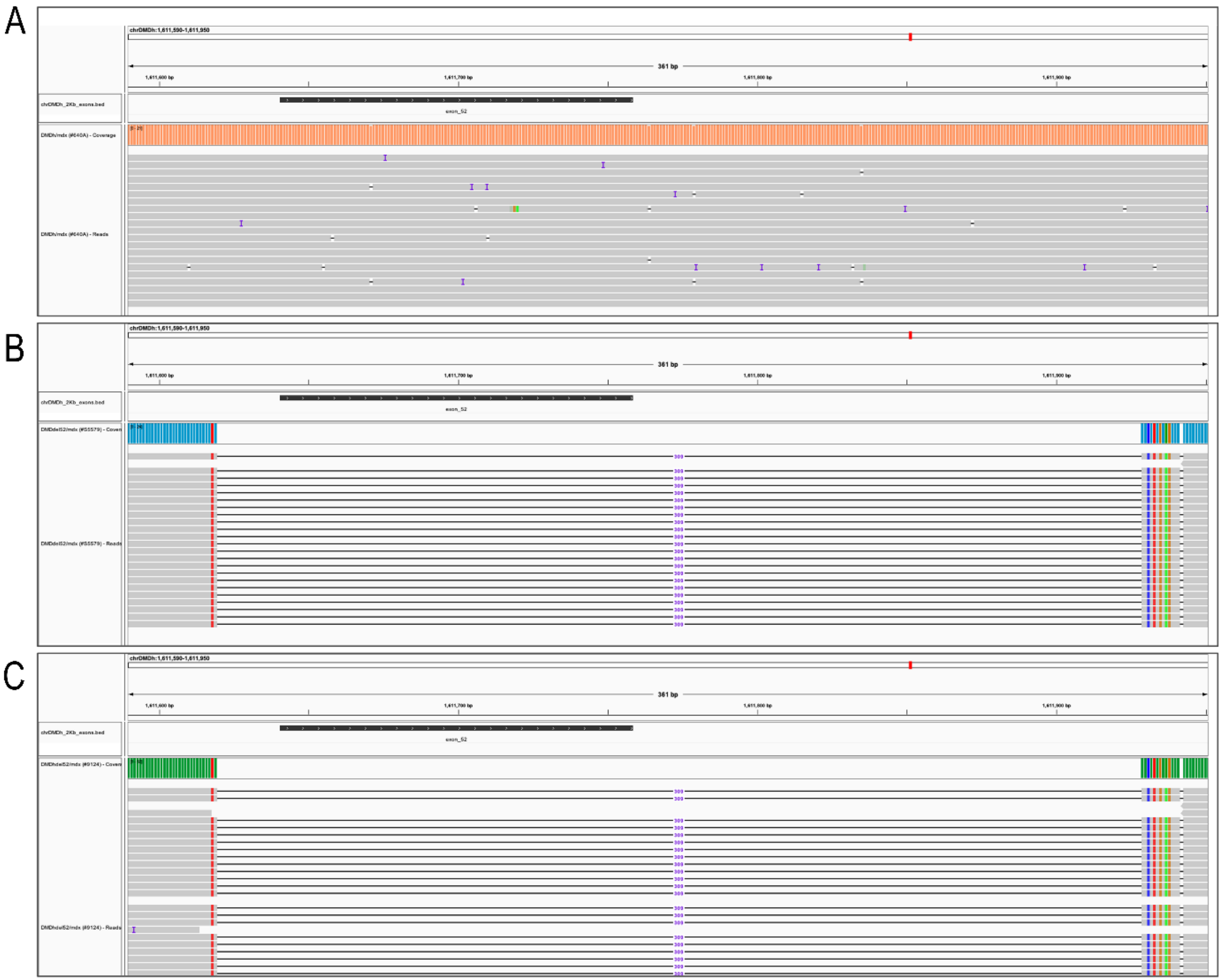

Disease Models & Mechanisms • Supplementary information

Fig. S3. IGV browser snapshot showing the coverage and reads alignment around the annotated exon 52 from the isoform Dp427m for the h*DMD* gene. Minimap2 alignment results were used for the structural variation analysis (A) hDMD/*mdx* heterozygous (sample #640A) showing exon 52 (black solid bar), the coverage track (orange), and a view of the read alignment (grey). (B) hDMDdel52/*mdx* heterozygous (sample #S5579) showing exon 52 (black solid bar), the coverage track (blue), and a view of the read alignment (grey). Reads show a deletion of 309 bp, spanning exon 52 and the adjacent intronic sequences. Mismatches between the reads sequences to the reference are colored on the reads. (C) hDMDdel52/*mdx* homozygous (sample #9124) showing exon 52 (black solid bar), the coverage track (green), and a view of the read alignment (grey). Reads show a deletion of 309 bp, spanning exon 52 and the adjacent intronic sequences. Mismatches between the reads sequences to the reference are colored on the reads.

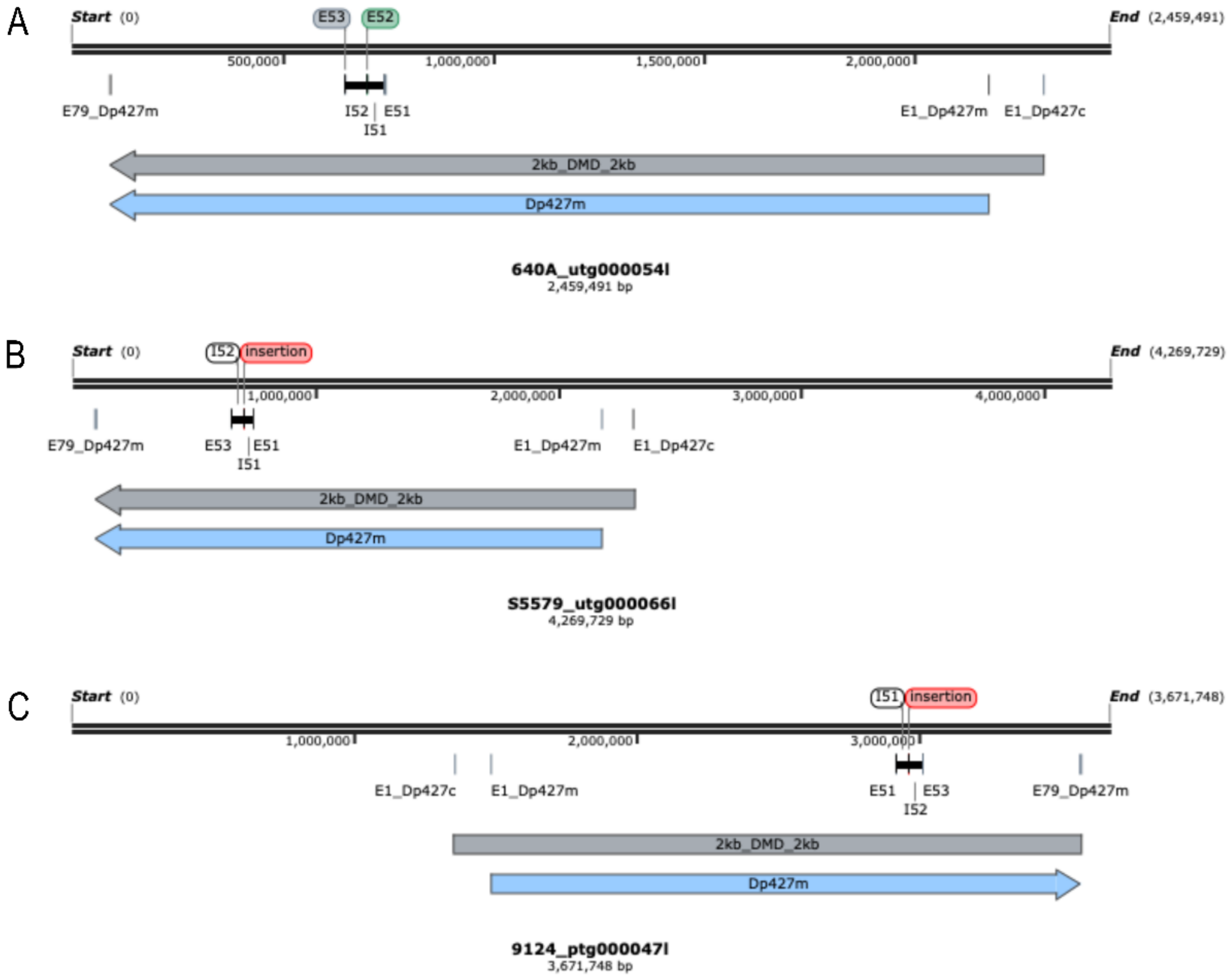

Fig. S4. *De novo* assembly of the *DMD* transgene from long-read sequencing. Assembled contigs from *de novo* genome assembly that were identified as containing the full human *DMD* transgene, including 2 kb of flanking region (arrow in gray; 2kb_DMD_2kb) from the most upstream exon (E1_Dp427c) and most downstream annotated exons (E79_Dp427m). The region spanning the main isoform expressed in muscle is shown as a blue arrow (Dp427m). (A) A *de novo* genome assembled contig (utg000054l) of 2,459,491 bp was obtained from the hDMD/*mdx* mice (#640A). The contig includes the full sequence of the *hDMD* gene, including all the exons for the MANE transcript (i.e., NCBI RefSeq NM_004006.3;

Disease Models & Mechanisms • Supplementary information

ENSMBL transcript id ENST00000357033). (B) A *de novo* genome assembled contig (utg000066l) of 4,269,729 bp was obtained from the hDMDΔ52/*mdx* mice (#S5579). The contig includes all exons for the MANE transcript except for exon 52. The small 16-bp insertion between introns 51 and 52 is present and shown in red between introns 51 and 52. (C) A *de novo* genome assembled contig (ptg000047l) of 3,671,748 bp was obtained from the hDMDΔ52/*mdx* mice (#9124). The contig includes all the exons for the MANE transcript except for exon 52. The small 16-bp insertion between introns 51 and 52 is present and shown in red between introns 51 and 52.

Disease Models & Mechanisms • Supplementary information

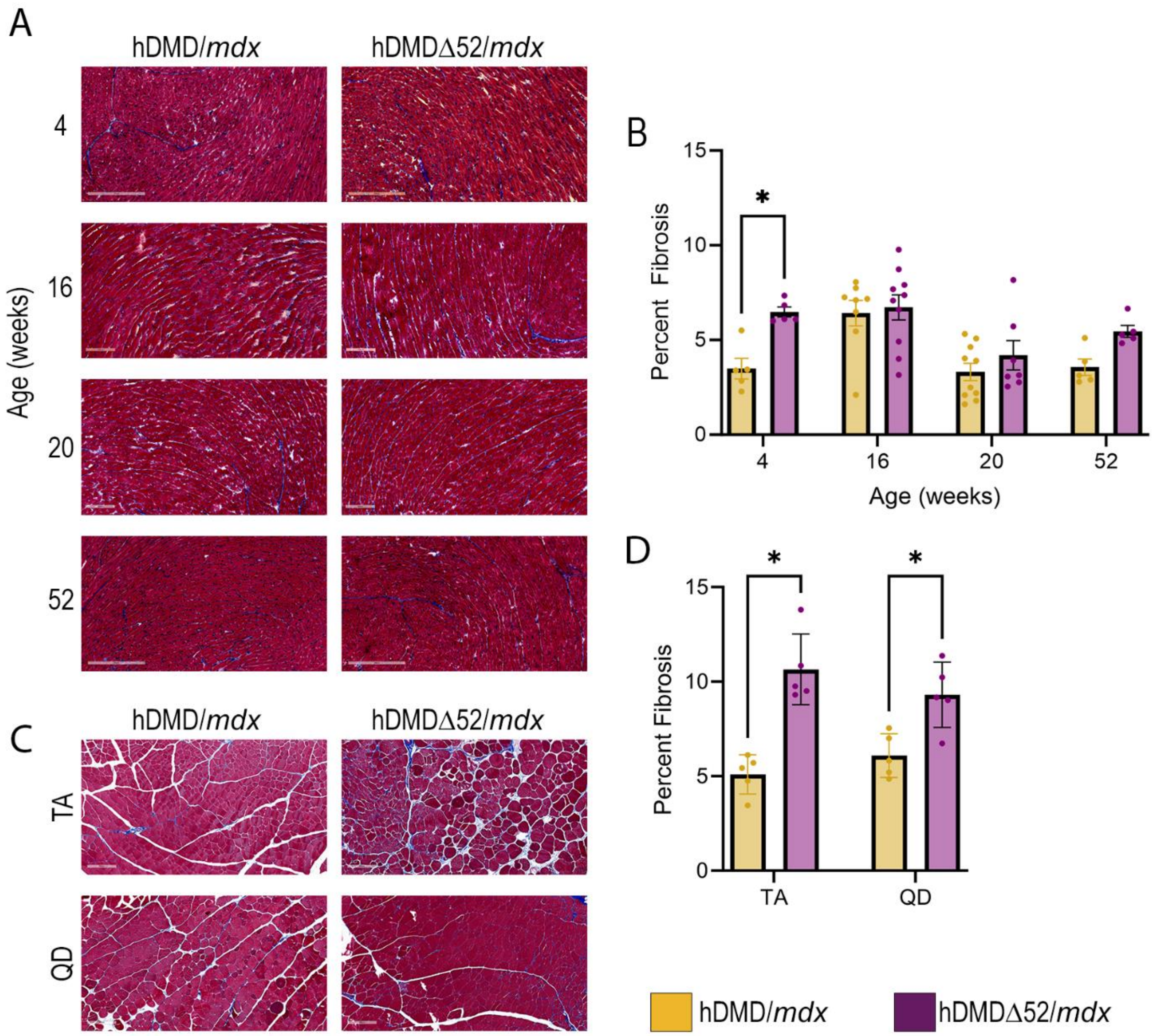

Fig. S5. Histological analysis shows a small increase in fibrosis at 4 weeks of age but no difference at older ages. Masson's trichrome staining of the heart at 4, 16, 20, and 52 weeks of age with quantification. (A) Representative images of Masson's Trichrome staining of heart tissues from hDMD/*mdx* and hDMDΔ52/*mdx* mice at the indicated ages. (B) Quantification of percent fibrosis in hDMD/*mdx* and hDMDΔ52/*mdx* mice. *$p<0.05$, two-way ANOVA. (C) Representative images of Masson's Trichrome staining of tibialis anterior (TA) and quadriceps (QD) muscles from hDMD/*mdx* and hDMDΔ52/*mdx* mice at 18 weeks of age. (D) Quantification of percent fibrosis in hDMD/*mdx* and hDMDΔ52/*mdx* mice. *$p<0.05$, Unpaired t-test. Data represent n=5-10 individual animals per group.

Disease Models & Mechanisms • Supplementary information

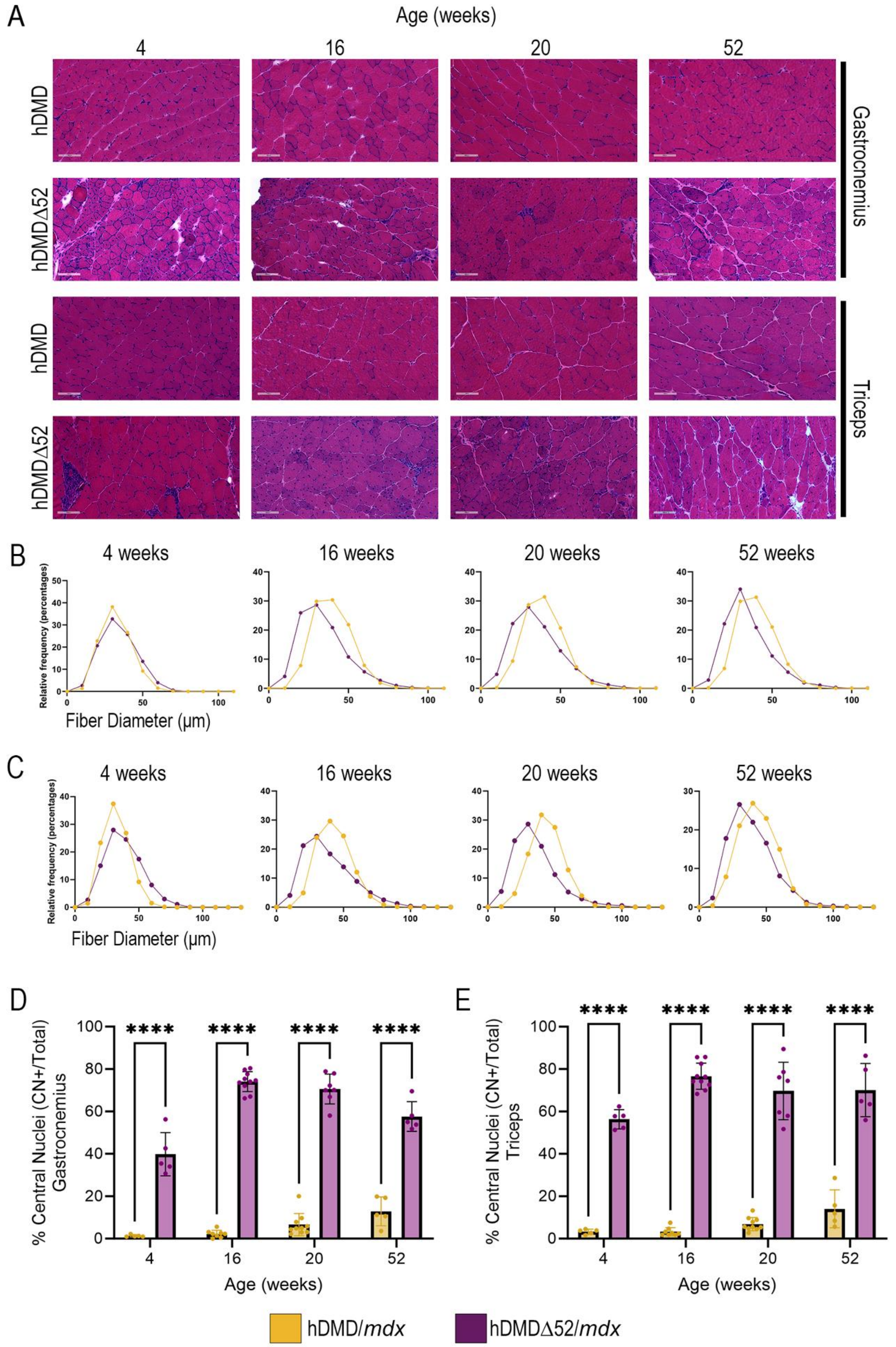
A
Age (weeks)
4
16
20
52
hDMD
hDMDΔ52
hDMD
hDMDΔ52
Gastrocnemius
Triceps
B
4 weeks
16 weeks
20 weeks
52 weeks
Relative frequency (percentages)
Fiber Diameter (µm)
C
4 weeks
16 weeks
20 weeks
52 weeks
Relative frequency (percentages)
Fiber Diameter (µm)
D
% Central Nuclei (CN+/Total) Gastrocnemius
Age (weeks)
****
E
% Central Nuclei (CN+/Total) Triceps
Age (weeks)
****
hDMD/mdx
hDMDΔ52/mdx

Fig. S6. Histological analysis reveals dystrophic phenotypes in the hDMDΔ52/*mdx* mice in left GAS and TRI muscles. (A) Representative images of H&E staining in the left GAS and TRI muscles of hDMD/*mdx* and hDMDΔ52/*mdx* mice at the indicated ages. (B, C) Fiber diameter in the left (B) GAS and (C) TRI muscles. (D, E) Central nuclei in the left (D) GAS and (E) TRI muscles. $^{****}p<0.0001$, one-way ANOVA. Data represent n=5-10 individual animals per group. GAS, gastrocnemius muscle; TRI, triceps muscle.

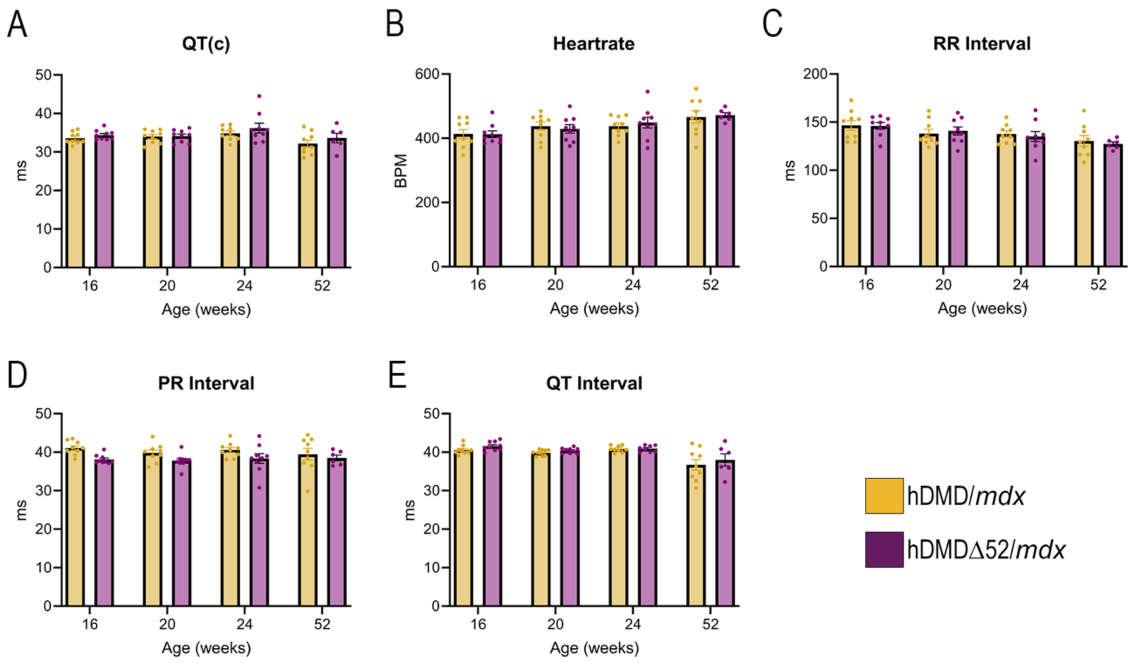

Fig. S7. Further characterization of heart function in hDMDΔ52/*mdx* mice. (A) Mean Qt(c) interval. (B) Mean heart rate. (C) Mean RR interval. (D) Mean PR interval. (E) Mean QT interval. No significant differences were observed by two-way ANOVA. Data represent n=6-10 individual animals per group.

Disease Models & Mechanisms • Supplementary information

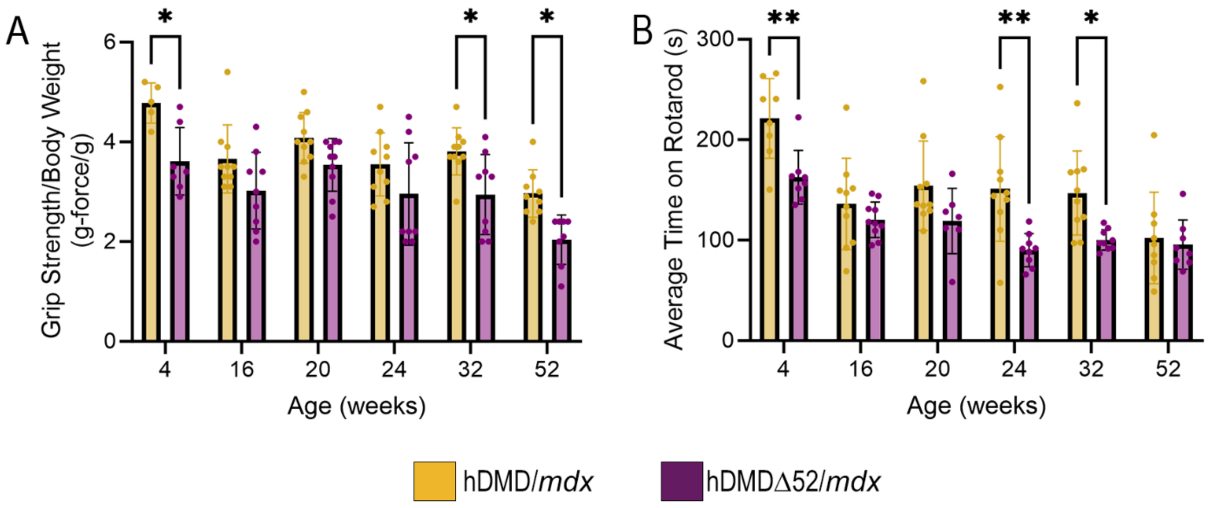

Fig. S8. hDMDΔ52/*mdx* mice display functional deficits. (A) Grip strength normalized to body weight at the indicated ages for hDMD/*mdx* and hDMDΔ52/*mdx* mice. (B) Time on rotarod at the indicated ages for hDMD/*mdx* and hDMDΔ52/*mdx* mice. *p<0.05, **p<0.01, two-way ANOVA. Data represent n=6-10 individual animals per group.

Disease Models & Mechanisms • Supplementary information

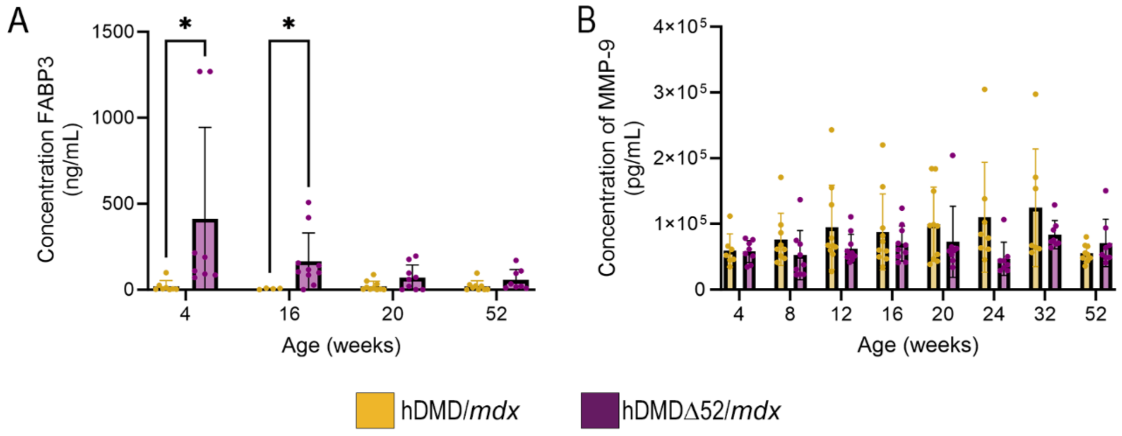

Fig. S9. Serum biomarkers of muscle and cardiac injury are elevated in hDMDΔ52/*mdx* mice. (A, B) Quantification of (A) FABP3 and (B) MMP-9. *$p<0.05$, Mann-Whitney test for nonparametric data. Data represent n=6-10 individual animals per group. FABP3, fatty acid binding protein 3; MMP-9, matrix metalloproteinase 9.

Disease Models & Mechanisms • Supplementary information

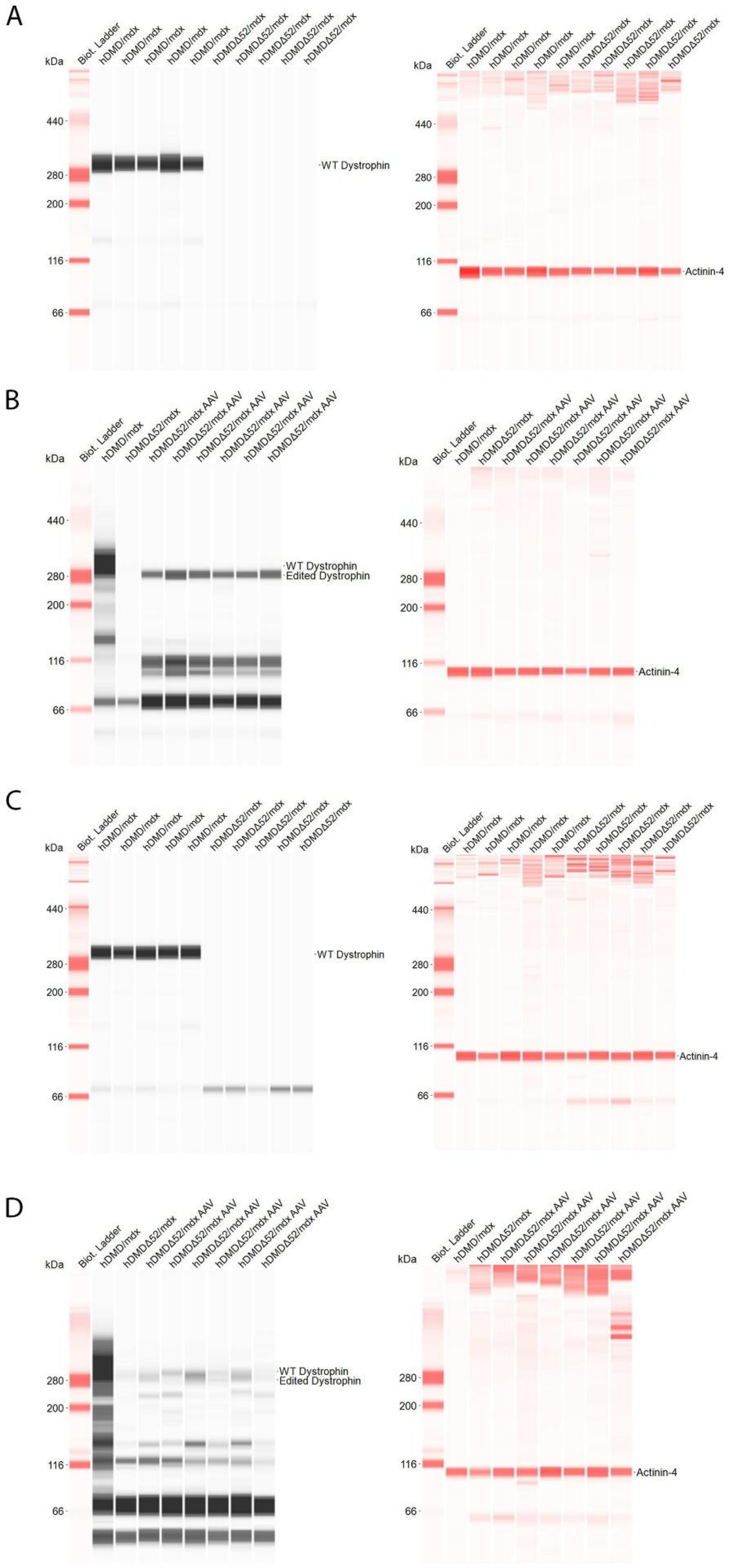

Disease Models & Mechanisms • Supplementary information

Fig. S10. Representative images of Jess Automated Western Blot protein quantification. (A) Dystrophin restoration and actinin control expression in hDMD/*mdx* and hDMDΔ52/*mdx* animals in the heart. Five animals from each group are shown. (B) Dystrophin restoration and actinin control expression in the hearts of hDMD/*mdx,* hDMDΔ52/*mdx,* and AAV-treated hDMDΔ52/*mdx* animals. One representative animal from each control group and six treated animals are shown. (C) Dystrophin restoration and actinin control expression in the TA muscle of hDMD/*mdx* and hDMDΔ52/*mdx* animals. Five animals from each group are shown. (D) Dystrophin restoration and actinin control expression in hDMD/*mdx,* hDMDΔ52/*mdx,* and AAV-treated hDMDΔ52/*mdx* animals in the TA muscle. One animal from each control group and six treated animals are shown. TA, tibialis anterior.

Table S1. Quality Control metrics for whole genome long-sequence data. Statistics for the whole genome sequence data for each of the four lines are presented. The table includes the total circular consensus sequencing (CCS) reads for each sample and the N50 metric (i.e., N50 is the read length at which 50% of the bases are in reads longer than this length).

Sample ID	Line	Genotype of *hDMD* allele	Sex	CCS reads	Number of CCS bases	CCS read length	N50
**640A**	hDMD/*mdx*	heterozygous	Male	4,672,852	71,851,660,183	15,376.404	16,562
**S5579**	hDMDΔ52/*mdx*	heterozygous	Male	4,103,176	65,459,355,745	15,953.339	17,567
**9124**	hDMDΔ52/*mdx*	homozygous	Female	4,992,454	68,012,873,197	13,623.135	15,066
**S9002**	*mdx*	NA	Male	4,355,932	63,318,373,627	14,536.125	15,472

Table S2. Reads mapped to determine location of chromosomal insertion. Whole genome sequencing reads aligned to the Hygromycin cassette sequence and to the yeast genome (as a proxy for the YAC sequence) and the further mapping of those reads against the mouse genome. The final column shows the reads located in the band 5qG2 that are indicative of the transgene insertion location.

Sample ID	Line	Genotype for the *DMD* allele	Sex	Total reads mapped (HygroR)	Total reads mapped (Yeast)	Total reads mapped (Mouse)	Reads mapped chr5:133,745,208-133,829,563
**640A**	hDMD/*mdx*	heterozygous	Male	24	25148	3915	18
**S5579**	hDMDΔ52/*mdx*	heterozygous	Male	51	47027	5493	29
**9124**	hDMDΔ52/*mdx*	homozygous	Female	29	22227	2919	16
**S9002**	*mdx*	NA	Male	0	178	178	0

Disease Models & Mechanisms • Supplementary information

Table S3. Reads mapped to specific putative *DMD* sequences. Reads containing the full sequence of several exons as annotated for the isoform Dp427m and the sequence of the junction between introns 51 and 52 with a 16-bp insertion for all the transgenic lines identified via Sanger sequencing.

	**Reads aligned to *hDMD* +/- 2kb**		
	**hDMD/*mdx* (het) 640A**	**hDMDdel52/*mdx* (het) S5579**	**hDMDdel52/*mdx* (homo) 9124**
**Exon_8**	28	21	55
**Exon_47**	31	29	49
**Exon_51**	19	25	50
**51_(+16bp)_52_junction**	0	20	45
**Exon_77**	27	24	50

Table S4. Study design.

	**Timepoint (weeks)**							
	**4**	**8**	**12**	**16**	**20**	**24**	**32**	**52**
**Functional assay**								
Grip force*	×			×	×	×	×	×
Rotarod	×			×	×	×	×	×
Physiology (TA)	×			×	×			×
Serum biomarker quantification**	×	×	×	×	×	×	×	×
ECG				×	×	×		×
**Histology**								
H&E	×			×	×			×
MTRI	×			×	×			×

Note- hDMD/*mdx* (n=5-10 per timepoint) and hDMDΔ52/*mdx* (n=5-10 per timepoint)
*Measured at least 3 days before the other tests.
**Serum was collected prior to any other outcomes performed at the same age.
ECG, electrocardiogram; H&E, hematoxylin and eosin; MTRI, Masson's trichrome; TA, tibialis anterior

Disease Models & Mechanisms • Supplementary information
